# Axonal connections between S1 barrel, M1, and S2 cortex in the newborn mouse

**DOI:** 10.3389/fnana.2023.1105998

**Published:** 2023-01-25

**Authors:** Levente Gellért, Heiko J. Luhmann, Werner Kilb

**Affiliations:** Institute of Physiology, University Medical Center of the Johannes Gutenberg University Mainz, Mainz, Germany

**Keywords:** cortical development, newborn mouse, subplate, sensorimotor integration, barrel field, motor cortex, secondary somatosensory cortex, DiI dye

## Abstract

The development of functionally interconnected networks between primary (S1), secondary somatosensory (S2), and motor (M1) cortical areas requires coherent neuronal activity *via* corticocortical projections. However, the anatomical substrate of functional connections between S1 and M1 or S2 during early development remains elusive. In the present study, we used *ex vivo* carbocyanine dye (DiI) tracing in paraformaldehyde-fixed newborn mouse brain to investigate axonal projections of neurons in different layers of S1 barrel field (S1Bf), M1, and S2 toward the subplate (SP), a hub layer for sensory information transfer in the immature cortex. In addition, we performed extracellular recordings in neocortical slices to unravel the functional connectivity between these areas. Our experiments demonstrate that already at P0 neurons from the cortical plate (CP), layer 5/6 (L5/6), and the SP of both M1 and S2 send projections through the SP of S1Bf. Reciprocally, neurons from CP to SP of S1Bf send projections through the SP of M1 and S2. Electrophysiological recordings with multi-electrode arrays in cortical slices revealed weak, but functional synaptic connections between SP and L5/6 within and between S1 and M1. An even lower functional connectivity was observed between S1 and S2. In summary, our findings demonstrate that functional connections between SP and upper cortical layers are not confined to the same cortical area, but corticocortical connection between adjacent cortical areas exist already at the day of birth. Hereby, SP can integrate early cortical activity of M1, S1, and S2 and shape the development of sensorimotor integration at an early stage.

## Introduction

In rodents, the whisker to barrel-cortex pathway represents an important sensory modality (for review see Feldmeyer et al., 2013; Staiger and Petersen, 2021). Due to its precise topographic organization throughout the processing pathway and the possibility of defined sensory stimulation, it also represents a classical model system to unravel sensory processing and perception (Woolsey and Van der Loos, 1970; Brecht, 2007; Petersen, 2007; Stüttgen and Schwarz, 2018). In adult rodents, intense interactions between primary somatosensory cortex barrel field (S1Bf), secondary somatosensory (S2), and motor cortex (M1) are necessary for adequate detection and perception of tactile stimuli (Fassihi et al., 2017; Petersen, 2019), and reciprocal connections of S1Bf with M1 and S2 cortices underlie whisker-related sensorimotor integration (Mao et al., 2011; Chen et al., 2013; Yamashita et al., 2013; Minamisawa et al., 2018).

A variety of studies demonstrated that spontaneous oscillatory activity and evoked cortical responses during pre- and early postnatal stages are essential for the development of functional connectivity between related cortical areas, like, e.g., primary and secondary sensory areas (Moreno-Juan et al., 2017, Anton-Bolanos et al., 2019, for reviews see Yamamoto and Lopez-Bendito, 2012, Leighton and Lohmann, 2016, Luhmann et al., 2016, Luhmann and Khazipov, 2018, Molnar et al., 2020, and Martini et al., 2021). While evoked responses, but also part of spontaneous activity, are generated in the periphery or in subcortical nuclei and forwarded to the corresponding cortical area (Hanganu et al., 2006; Ackman et al., 2012), a considerable fraction of spontaneous activity transients are generated within neocortical circuits (Garaschuk et al., 2000; Khazipov and Luhmann, 2006; Blankenship and Feller, 2010; Luhmann et al., 2016). This spontaneous and evoked oscillatory activity spans areas with distinct functional role (Brockmann et al., 2011, Yang et al., 2013, Del Rio-Bermudez and Blumberg, 2018, for reviews see Garaschuk et al., 2000, Khazipov and Luhmann, 2006, and Martini et al., 2021). The establishment of the functional connectivity between S1 and M1 was previously demonstrated, showing that M1 is involved in somatosensory information processing during early development [between postnatal day 3 and 12 (P3-12)] in the rat (An et al., 2014; Dooley and Blumberg, 2018; Gomez et al., 2021). Functional connections between S1 and S2 appear around the end of the first postnatal week (Cai et al., 2022). No information is currently available on anatomical and functional connectivity between S1, S2, and M1 in mouse at the earliest postnatal stages. In addition, the anatomical basis of corticocortical interactions between S1 and S2 during early postnatal development remains to a large extend elusive.

During perinatal stages the transient cortical subplate (SP) serves as an essential element for the transmission of peripheral information to the developing cortex (Viswanathan et al., 2017, Wess et al., 2017, for review see Kanold and Luhmann, 2010). A disturbance in the SP can result in persistent dysfunction of the mature cortex (Tolner et al., 2012; Nagode et al., 2017; Sheikh et al., 2019). The SP receives glutamatergic inputs from the thalamus (Friauf et al., 1990; Zhao et al., 2009), as well as glutamatergic and GABAergic inputs from intracortical sources, including horizontal connections within the SP (Hanganu et al., 2002; Hirsch and Luhmann, 2008; Viswanathan et al., 2012). Furthermore, SP cells are electrically coupled with cortical plate (CP) and neighboring SP neurons *via* gap junctions (Dupont et al., 2006; Moore et al., 2014). SP neurons project to the developing cortical layers forming a transient glutamatergic and GABAergic network (Finney et al., 1998; Hanganu et al., 2009; Myakhar et al., 2011; Viswanathan et al., 2012). Recently, long-range projections of SP neurons were described in the mouse. SP cells in S1 were found to send long-range unilateral axonal projections to M1 already at P2 (Tiong et al., 2019), while backlabeled neocortical SP cells were found upon tracer injection into distant ipsilateral areas or corresponding contralateral areas, with few GABAergic neurons contributing to these connections (Boon et al., 2019). Thus the intense local connectivity and the long-range corticocortical projections of SP neurons may underlie the generation and global synchronization of oscillatory activity in the immature rodent and human cortex (for reviews see Kostović, 2020 and Molnar et al., 2020). Through this local and remote connectivity, the SP provides an early integrative hub, which is essential for the development of the cortical network cortex (for review see Luhmann et al., 2009 and Luhmann et al., 2018).

Based on these properties of the SP, we hypothesize that the SP may organize axonal projections between M1, S1Bf, and S2 already at P0, thereby integrating neuronal activity between these cortical areas during the earliest stage of postnatal development. We investigated the distant projections between M1, S1Bf, and S2 through SP by *ex vivo* carbocyanine dye (DiI) tracing in paraformaldehyde-fixed newborn mouse brain tissue as well as by multi-shank silicon probe recordings. These experiments demonstrate for the first time that in the neocortex of newborn mice at P0 (i) neurons in M1 and S2 send projections through the SP of S1Bf, that (ii) neurons in S1Bf send projections through the SP of M1 and S2 and (iii) that these projections form weak but functional synaptic networks in the early postnatal neocortex.

## Materials and methods

### Animals

All experiments were conducted in accordance with National and European (86/609/EEC) laws for the use of animals in research and were approved by the local ethical committee (Landesuntersuchungsamt Rheinland-Pfalz). All efforts were made to minimize the number of animals and their suffering. Offspring from timed-pregnant C57BL/6NRj wild-type mice (Janvier labs) were used for all experiments. The timed-pregnant dams were kept in a 12/12 day/night cycle, had access to food and water *ad libitum*, and were checked for delivery every day.

### Carbocyanine dye labeling and tissue processing

P0 (<24 h after birth) mouse pups were decapitated, brains were carefully removed from the skull and immersion fixed in buffered 4% paraformaldehyde (PFA) for 48 h at 4°C. Brains were then rinsed in phosphate buffered saline (PBS), subsequently embedded in 3% PBS-agarose and sectioned coronally with a vibratome (Leica VT 1000S, Leica Biosystems, Wetzlar, Germany) until reaching a plane with a co-appearance of M1, S1Bf, and S2. The fluorescent nuclear marker (SYTOX™ Green, Invitrogen, Waltham, MA, USA) was topically applied onto the cut surface of the remaining brain block to identify cortical areas and the SP under a fluorescence stereomicroscope (Wild Heerbrugg, Switzerland) that was equipped with an adequate filter and illumination set (Nightsea, ScienceLabs; Lexington, KY, USA). The slice plane and the cortical regions were identified according to an immature mouse brain atlas (Paxinos, 2007). The course of the lateral ventricle and cingulum, tips of the internal capsule and the fimbria were used for orientation. The remaining part of the immersion-fixed brain was used as sample. Using a tungsten wire fixed to a manual micromanipulator (WPI, Sarasota, FL, USA), a single DiI crystal (1,10-didodecyl-3,3,30,30-tetramethyl-indocarbocyanine perchlorate; ≈50 μm in diameter; Thermo Fischer, Waltham, MA, USA) was placed under stereomicroscope control into the SP-L6 border of either S1Bf, M1, or S2, respectively (Supplementary Figure 1). Samples were incubated in PBS-Azide (0.05%) for 4 weeks at 37°C. Following the incubation, 350 μm thick slices were re-sectioned from the brain block starting at the sectioned surface. Subsequently, NeuN immunohistochemistry (see Section “Immunohistochemistry on DiI injected samples”) was performed on these slices before clearing with the DiI-compatible SeeDB method optimized for neonatal mouse brain (Ke et al., 2013). After clearing, slices were placed onto microscope slides, embedded in the clearing solution, coverslipped, and sealed with dental silicone (Picodent Twinsil^®^, Wipperfürth, Germany).

### Immunohistochemistry on DiI injected samples

To better visualize the SP for surface fitting and cortical thickness estimation we performed NeuN immunohistochemistry following the incubation of the samples with DiI. To keep the DiI labeling intact we applied a low concentration of the cholesterol-specific detergent digitonin (Thermo Fischer, Waltham, MA, USA) (Matsubayashi et al., 2008). Briefly, free-floating 350 μm thick samples were blocked in a blocking solution (PBS-Azide, 2% normal donkey serum, 25 μg/ml Digitonin) for 3 h at room temperature, followed by an overnight exposure to the primary antibody in the blocking solution at 4°C (rabbit anti-NeuN, 1:1000, Abcam, Cambridge, UK; ab177487). Next day, samples were incubated in DAPI (Thermo Fischer, Waltham, Massachusetts, USA) supplemented blocking solution containing the secondary antibody (1:200; donkey anti-rabbit Alexa 647; Jackson ImmunoResearch, Cambridge, UK; 711-605-302) at room temperature for 3 h.

### Imaging and data analysis

**FIGURE 1:**
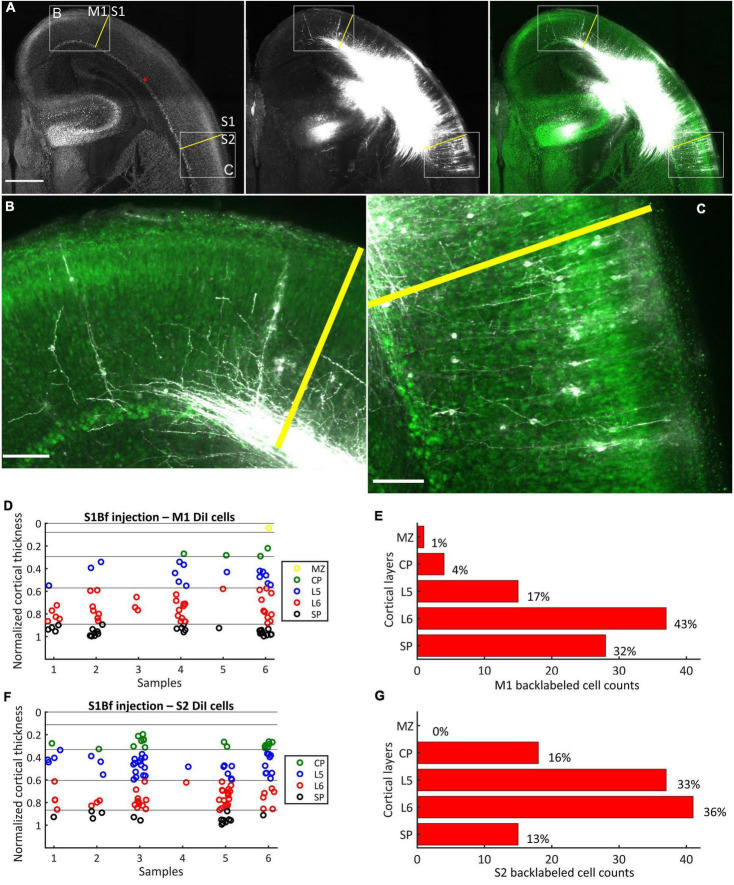
DiI labeling pattern and localization of DiI backlabeled cells in M1 and S2 on six S1Bf injected samples. **(A)** Representative photomicrographs showing the DiI labeling pattern in M1 and S2 cortices of S1Bf injected sample. Image was prepared as a maximum intensity projection of selected stacks of 350 μm thick samples. Left panel: NeuN counterstain of DiI injected sample. Red asterisk indicates the DiI injection site in S1Bf. Yellow lines show border between M1 and S1 (upper left) and between S1 and S2 (lower right) as estimated from reference points in the developing mouse brain atlas (Paxinos, 2007). Middle panel: DiI tracer spreading pattern toward M1 and S2 cortices. Right panel: Composite image of the DiI signal and the pseudocoloured NeuN signal showing the DiI spread toward M1 and S2 cortices. **(B)** Representative M1 area, as indicated in panel **(A)** (rectangle a), showing backlabeled cell distribution across cortical layers. **(C)** Representative S2 area as indicated in panel **(A)** (rectangle b), showing backlabeled cell distribution across cortical layers. Scale bars are 500 μm **(A)** and 100 μm **(B,C)**. **(D,F)** Scatter plots showing the localization of manually detected somata of backlabeled cells through the normalized cortical thickness in M1 **(D)** and in S2 **(F)** in 6 S1Bf injected samples. Zero level at the y axis refers to the pial surface, level 1 indicates the SP-white matter boundary. **(E,G)** Quantification of observed counts of backlabeled cells in M1 **(E)** and in S2 **(G)**.

**FIGURE 2:**
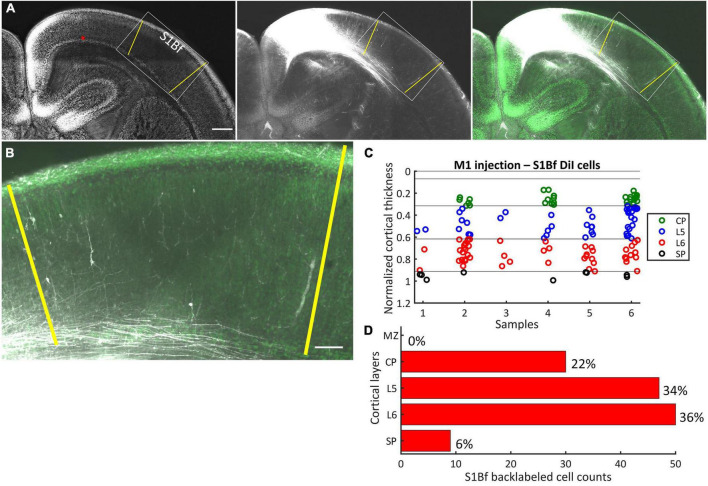
DiI labeling pattern and localization of DiI backlabeled cells in S1Bf on six M1 injected samples. **(A)** Representative photomicrograph showing the DiI labeling pattern in S1Bf on M1 injected sample. Image was prepared as a maximum intensity projection of selected stacks of 350 μm thick sample. Left panel: NeuN counterstain of DiI injected sample. Red asterisk indicates the DiI injection site in M1. Yellow lines demark S1Bf from the adjacent primary somatosensory cortices, according to reference points in the developing mouse brain atlas (Paxinos, 2007). Middle panel: DiI tracer spreading pattern toward S1Bf. Right panel: Composite image of the DiI signal and the NeuN signal showing the DiI spread toward S1Bf. **(B)** Representative S1Bf area, as indicated in the rectangle on panel **(A)**, showing backlabeled cell distribution across cortical layers. Scale bars are 500 μm **(A)** and 100 μm **(B)**. **(C)** Scatter plot showing the localization of manually detected somata of backlabeled cells through the normalized cortical thickness in six S1Bf injected samples. Zero level at the y axis refers to the pial surface, level 1 indicates the SP-white matter boundary. **(D)** Quantification of observed counts of backlabeled cells in M1.

**FIGURE 3:**
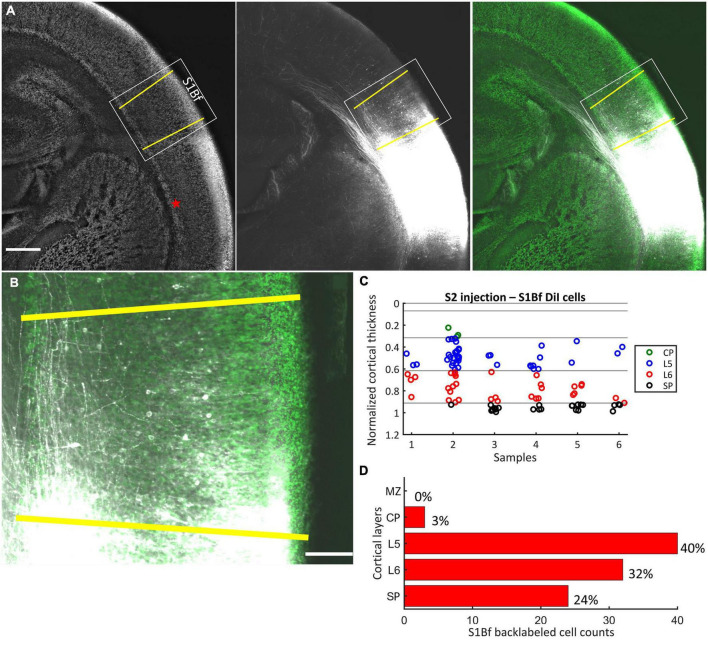
DiI labeling pattern and localization of DiI backlabeled cells in S1Bf on six S2 injected samples. **(A)** Representative photomicrograph showing the DiI labeling pattern in S1Bf on S2 injected sample. Image was prepared as a maximum intensity projection of selected stacks of 350 μm thick sample. Left panel: NeuN counterstain of DiI injected sample. Red asterisk indicates the DiI injection site in S2. Yellow lines demark S1Bf from the adjacent primary somatosensory cortices, according to reference points in the developing mouse brain atlas (Paxinos, 2007). Middle panel: DiI tracer spreading pattern toward S1Bf. Right panel: Composite image of the DiI signal and the NeuN signal showing the DiI spread toward S1Bf. **(B)** Representative S1Bf area, as indicated in the rectangle on panel **(A)**, showing backlabeled cell distribution across cortical layers. Scale bars are 500 μm **(A)** and 100 μm **(B)**. **(C)** Scatter plot showing the localization of manually detected somata of backlabeled cells through the normalized cortical thickness in six S1Bf injected samples. Zero level at the y axis refers to the pial surface, level 1 indicates the SP-white matter boundary. **(D)** Quantification of observed counts of backlabeled cells in S1Bf.

Samples were imaged with a VisiScope Spinning Disc confocal microscope (10× dry objective, 2.5 μm Z steps, Visitron Systems GmbH, Puchheim, Germany). A confocal tiled scan was performed to image the area of M1, S1Bf, and S2, respectively. Tiles were then stitched with the built-in stitching plugin of Fiji (Preibisch et al., 2009). To create 3D segments for manual cell counting, the Segmentation editor ImageJ plugin was used (Gul-Mohammed et al., 2014). Medio-lateral boundaries of M1, S1Bf, and S2 cortical areas were drawn manually on every 20th stack and were interpolated. All analyses are based on the identification of neuronal somata, that demonstrated obvious DiI fluorescence due to retrograde fluorophore diffusion from the DiI injection site. Backlabeled cells were counted manually in the 3D segments and stack position of each detected cell was defined by visual control. For further analysis x, y, z centroid coordinates of the backlabeled cells were used. Local thickness of the cerebral cortex and the disposition of backlabeled cells through cortical layers were defined by custom-written MatLab routines based on Euclidean distance estimation from the pial and SP surfaces, respectively. Cerebral pial and white matter-gray matter boundary were drawn manually and 3D surfaces were created with the MatLab “surffit” function. Local thickness of the cortex at every backlabeled cell was defined as distance between pial surface and the lower SP surface and the position of the corresponding backlabeled cell was normalized to this value. Backlabeled cells in M1 and S2 were counted on the same samples after injection in S1Bf (see Figure 1A). Backlabeled cells in S1Bf after injections in M1 (see Figure 2A) or S2 (see Figure 3A) were counted on separate samples.

### Estimation of neuron number

To estimate the proportion of neurons across cortical layers, standard NeuN immunohistochemistry was performed on separate P0 mouse brain slices. Briefly, PFA-fixed brains were immersed in 30% sucrose-PBS solution for cryoprotection until probes were sank onto the bottom of incubation tubes (overnight at 4°C). Slices of 12 μm thickness were cut on a freezing microtome (Leica CM1325, Wetzlar, Germany) and processed on-slide thereafter. Samples were permeabilized and blocked in a blocking solution (PBS-Azide, 5% normal donkey serum, 0.4% Triton™ X-100) for 1 h at room temperature, following an overnight exposure to the primary antibody solution at 4°C (rabbit anti-NeuN 1:1000, Abcam, Cambridge, UK; dissolved in PBS, 1% normal donkey serum). On the next day, samples were incubated in the secondary antibody (donkey anti-rabbit Cy3, Jackson ImmunoResearch 711-165-152, Cambridge, UK; dissolved in PBS, 1% normal donkey serum supplemented with DAPI) at room temperature for 2 h. Samples were then mounted in ProLong™ Gold antifade mountant (Waltham, MA, USA) and coverslipped. The whole dorso-ventral thickness of M1, S1Bf, and S2 cortices, respectively, were tile-imaged in a width of one-field-of-view of the 60X water immersion objective with adequate filter sets for DAPI and Cy3 on the Visiscope Spinning Disk confocal microscope. Tiles were stitched as described above.

The DAPI channel of the digital images were segmented with the Fiji Trainable Weka Segmentation plugin (Arganda-Carreras et al., 2017) and were binarized. At coordinates of logical zeros (background) of the DAPI image we set the gray-level value of NeuN images to zero. NeuN positive nuclei were then counted manually (Table 2). To assign cortical layer boundaries the following criteria were used: the marginal zone (MZ) was considered a thin, sparsely populated cell layer between DAPI labeled flat meningeal cells and NeuN positive dense cortical plate (CP) neurons. CP consisted of densely packed spindle-shape NeuN positive cells distinctive from layer 5 (L5) with large round NeuN positive cells. L6 cells showed smaller cell size and a relatively lower expression of the NeuN marker. Subplate (SP) layer could easily be delineated from the underlying white matter (WM) upon different cell structure and cell size. A high number of large, strongly NeuN positive cells with non-uniform morphology demarked the SP from the above L6 (Supplementary Figure 2). Thickness ratio from three animals was defined as summarized in Table 1.

**TABLE 1:** Relative thickness of cortical layers.

Cortical area	MZ	CP	L5	L6	SP
M1	0.079	0.215	0.276	0.320	0.107
S1Bf	0.070	0.245	0.301	0.263	0.088
S2	0.112	0.218	0.273	0.867	0.132

Layer boundaries were assigned on 12 μm thick coronal sections, stained for DAPI and NeuN in M1, S1Bf and S2 (Supplementary Figure 2). Relative layer thickness data were used for total neuron number estimations in each layer (Table 2) and for assigning the backlabeled cells to layers.

**TABLE 2:** Relative proportion of total neuron numbers in cortical layers.

Cortical area	MZ	CP	L5	L6	SP
M1	0.06	0.29	0.24	0.34	0.07
S1Bf	0.06	0.33	0.24	0.31	0.06
S2	0.08	0.32	0.22	0.30	0.09

NeuN positive nuclei were counted manually throughout the whole cortical thickness in 12 μm thick coronal sections of M1, S1Bf, and S2 (Supplementary Figure 2). Proportions were used to estimate expected backlabeled cell frequencies in the statistical analysis.

### Slice preparation and electrophysiological recordings

Newborn mouse pups (<24 h) were decapitated upon deep anesthesia (Ethrane, Abbot Laboratories, Wiesbaden, Germany). Whole-brain coronal slices including M1, S1Bf, and S2 were cut on a vibroslicer (Microm HM 650 V) at 400 μm thickness. Some slices were divided into two hemispheres. Slices were maintained for 1 h at room temperature in a storage chamber before transferring them into a submerged recording chamber (perfusion rate of 5 ml/min; 32°C). During preparation, slice maintenance and during electrode positioning, slices were maintained in artificial cerebrospinal fluid (standard ACSF) containing (in mM): 126 NaCl, 26 NaHCO_5_, 2.5 KCl, 2 CaCl_2_, 1 MgCl_2_, 1.25 NaH2PO_5_, and 10 D-glucose The pH of the solution was 7.4 after equilibration with 95% O_2_ and 5% CO_2_.

After electrode positioning spiking activity was evoked chemically by superfusing the slices with nominally Mg^2+^-free ACSF, containing (in mM): 126 NaCl, 26 NaHCO_5_, 2.5 KCl, 2 CaCl_2_, 0 MgCl_2_, 1.25 NaH2PO_5_, and 10 D-glucose, saturated with 95% O_2_ and 5% CO_2_, and supplemented with 50 μM 4-aminopyridine (4-AP; Sigma Taufkirchen, Germany) to promote spontaneous activity (Perreault and Avoli, 1989; Richter et al., 2010).

Extracellular signals were recorded with silicon probes (A4 × 8-A32, 32-channel multi-electrode array (MEA) in a 4-shank configuration, inter-shank distance of 200 μm, inter-channel distance of 100 μm; Neuronexus, Ann Arbor, MI, US.) at 20 kHz with USB-MEA256-System and MC_Rack software (Multi Channel Systems, Reutlingen, Germany). Raw signals were processed off-line. Multi-unit activity (MUA) was extracted from 200 to 5,000 Hz filtered raw signals by applying a threshold of five times the baseline standard deviation of a spike free interval (custom-written MatLab routines, MatLab software version: 7.7; Mathworks, Natick, MA, USA). Simultaneous recordings were obtained in two functionally distinct cortical areas. For this purpose, a pair of DiI coated 4-shank silicon probes were positioned in the slices in one of this two configurations: (i) M1 and S1Bf (six recordings in six slices, Supplementary Figure 3A) or (ii) S2 and S1Bf (five recordings in five slices, Supplementary Figure 3B). The ventral middle shank was positioned into the SP (SP shank hereafter) and the dorsal middle shank into the border of L5 and L6 (L5/6 shank hereafter). The ventral lateral shanks fall out of the cerebral cortex, the dorsal lateral ones fall into the CP MZ border and only rarely showed spiking. Electrical activity could be reliably recorded from four channels of these SP and L5/6 shanks, respectively. After recordings the slices were transferred into 4% PFA supplemented with DAPI for 48 h. DAPI and DiI fluorescent signal was imaged in μ-dish (Ibidi) with an epifluorescent microscope.

### Analysis of coincident spiking

To analyze the timing relation of spike trains within the presumable range of monosynaptic connection delay (Hanganu et al., 2002; Zhao et al., 2009; Myakhar et al., 2011), spike cross-correlograms (CCGs) were generated between reference and target channel pairs of the silicon probes. Electrodes in the area of backlabeled cells detected in the tracing study, were considered as reference electrodes. Electrodes in the SP (area of DiI crystal injection in the tracing study) were considered as target electrodes. To find above-chance coincident firing, surrogate spike data was generated (custom-written MatLab routines, MatLab software version: 9.5.0.944444). To preserve the original non-stationary firing pattern, joint interspike interval dithering (jisi-di) surrogate generation method was used, which is robust to avoid false positivity (Louis et al., 2010). As surrogate data did not show normal distribution, threshold was set at the 99 percentile of the 1,000 surrogate spike train.

### Coincident bursting analysis

Bursts were identified from the MUA spike time datasets as a sequence of at least three successive spikes with an interspike interval ≤300 ms. For the detection of coincident bursts, we performed a pairwise comparison between MUA spike time datasets from two electrode positions and identified bursts in which at least one spike of the burst in one electrode appeared within the bursting interval of the second electrode. The relative number of coincident bursts was calculated for each electrode pair by dividing the number of coincident bursts by the total number of bursts in the electrode with the lower burst activity. In order to analyze the temporal relation of mutual bursting activity we compared timing of the initial spikes of coinciding bursts. Ratio of the leading and lagging bursts were calculated. For each electrode pair the ratio of burst that lead or lag was calculated by normalizing the respective number to the total number of coinciding bursts in this electrode pair. All analyses were repeated for a surrogate dataset, consisting of 1,000 spike trains with a spike jittering by 750 ms (i.e., the approximated duration of a burst).

### Statistical analysis

For statistical comparison of backlabeled cells across cortical layers, chi-square goodness of fit test (chi2gof) was performed (IBM SPSS Statistics software, version 23; Armonk, NY, USA). The model investigates whether the observed proportion of backlabeled cells differs significantly from the proportion of all neurons within each cortical layer. Since estimating the total number of neurons was not feasible in our samples, the proportion of neuron number in cortical layers was determined (Table 2) in a 12 μm thick NeuN brain slices (see Section “Estimation of neuron number” in the Section “Materials and methods”). If a significant difference of the relative numbers of backlabeled neurons from the relative number of all neurons throughout the layers was found with the Chi2gof test, we checked layerwise if the relative number of backlabeled cells is significantly different from the relative neuron number using a binomial test (MatLab). For statistical analysis of the electrophysiological data the non-parametric Wilcoxon signed rank test (Systat 11, Systat software, Berkshire, UK) was used. The significance level α was set to 0.05 (*) and 0.01 (^**^).

## Results

Small DiI crystals were placed at the SP-L6 border either in M1 (Supplementary Figure 1A), S1Bf (Supplementary Figure 1B), or S2 (Supplementary Figure 1C) onto the cut surface of a trimmed brain block (see Section “Materials and methods” for details). After 4 weeks of incubation we analyzed the distribution of the somata of neurons with a clear DiI signal, which therefore must extend their axon through the DiI injection site (termed “backlabeled cells”). The majority of backlabeled cells showed pyramidal morphology, however, because of differences in the filling of the backlabeled cells with DiI and the uncertain morphology of neurons at this early developmental stage (Valverde et al., 1989; Luhmann et al., 1999; Gaïarsa et al., 2001), morphological classification of backlabeled cells in the cortical layers was not performed. We also did not further classify the morphological properties of the backlabeled cells in the SP (Hanganu et al., 2002; Hoerder-Suabedissen and Molnar, 2012). All backlabeled cells with clearly filled somata and processes were included in the analysis.

### S1Bf injections

After DiI injections at the SP-L6 border in S1Bf, somata of backlabeled cells were counted in M1 and S2 regions of the same sample (Figures 1A–C). In six analyzed samples we found in total 85 cells in M1 (Figure 1D) and 111 cells in S2 (Figure 1F). In both, M1 and S2 the highest proportion of backlabeled cells was found in L6: 43% (37/85 cells) in M1 and 36% (41/111) in S2, respectively. In L5 of S2 the proportion of backlabeled cells was 33% (37/111) while only 17% (15/85) of the backlabeled cells of M1 were located in L5. A similar tendency was found in the CP, where 16% (18/111) of the backlabeled cells were detected in the CP of S2 and only 4% (4/85) in the CP of M1. In contrast, in S2 only 13% (15/111) of the backlabeled cells were located in the SP, whereas in M1 32% (25/85) were located in the SP. Only one backlabeled cell was detected in the MZ of M1. Counts and relative occurrence of backlabeled cells across layers in M1 and S2 are depicted in Figures 1E, G. No backlabeled cells were detected in cingulate (medial to M1) and in insular (lateral to S2) cortices, suggesting that projections from these areas to the S1Bf injection site were absent in our samples. In summary, these results indicate, that both M1 and S2 are projecting toward the SP region of the S1Bf already at P0.

### M1 injections

After DiI injections at the SP-L6 border in M1, in total 136 somata of backlabeled cells (within six samples) were detected in S1Bf (Figures 2A–C). In contrast, no backlabeled cells were detected lateral to the S1Bf–primary somatosensory cortex upper lip boundary (lateral to S1Bf; Figure 2A). Thus, we can exclude axonal projections from S2 to the M1 DiI injection site. No backlabeled cells were detected in the MZ of S1Bf. Within S1Bf, the highest proportion of backlabeled cells (36%, corresponding to 50/136 cells) was detected in L6. A comparable proportion of backlabeled cells 34% (47/136) was detected in L5. In the CP, 22% (30/136) of the backlabeled cells were found, while a relatively low proportion of 6% (9/136) was found in the SP of S1Bf. Counts and relative occurrence of backlabeled cells across layers in S1Bf are depicted in Figure 2D. In summary, these results indicate, that neurons from the S1Bf project toward the SP region of M1 already at P0, but projections from S2 toward SP of M1 are not established at this developmental stage.

### S2 injections

After DiI injections at the SP-L6 border in S2, in total 99 somata of backlabeled cells in six samples were detected in the S1Bf (Figures 3A–C). Backlabeled cells were not detected medial to the S1Bf–primary somatosensory cortex forelimb boundary (lateral to S1Bf). This observation indicates that projections from M1 to the S2 DiI injection site can be excluded in our samples. Within S1Bf no backlabeled cells were detected in the MZ. The highest proportion (40%, corresponding to 40/99 cells) of backlabeled cells in S1Bf was detected in L5. A lower proportion of 32% (30/99) was found in L6. A rather low proportion (3%, corresponding to 3/99 cells) of backlabeled cells was detected in the CP. Finally, 24% (24/99) of the backlabeled cells were detected in the SP. Counts and relative occurrence of backlabeled cells across layers in S1Bf are depicted in Figures 3C, D. In summary, these results indicate, that neurons from the S1Bf project toward the SP region of S2 already at P0, but projections from M1 toward SP of S2 are not established in this developmental stage.

### Statistical comparisons

The massive differences in the number of neurons within the distinct layers must be taken into account to determine whether neurons from a particular cortical layer have a higher probability to project toward the DiI injection site. Therefore, we calculated the proportions of neuron number for all layers from NeuN-stained thin cortical slices. These proportions of all neurons were compared with the experimentally determined proportion of backlabeled cells for each layer and the significance of the difference was calculated with a binomial test. Since we found a lack of projections from the MZ toward the DiI injection site, with the exception of one single cell in the MZ of M1 in one single S1Bf injected sample (Figure 1D; sample 6), the MZ was excluded from statistical analysis.

**FIGURE 4:**
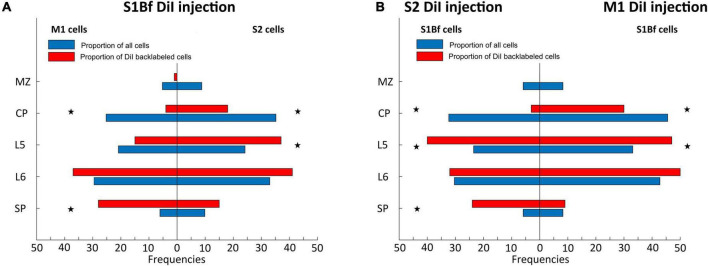
Statistical comparison of observed proportions of DiI backlabeled cell with the proportion of all neurons across cortical layers. **(A)** Proportions of backlabeled and all neurons within M1 (left panel) and S2 (right panel) on S1Bf injected samples. **(B)** Proportions of backlabeled and all neurons within S1Bf upon S2 (left panel) and M1 (right panel) injections. Asterisks indicate if the observed proportion of back-labeled cells differs significantly (*p* > 0.05) from the proportion of neurons in the respective layer.

This statistical evaluation revealed that in S1Bf injected samples, the proportion of backlabeled cells in the CP of M1 and S2 was significantly (*p* = 0.001 for M1; *p* = 0.001 for S2) lower than expected from the proportion of all neurons in this layer (Figure 4A). In L5 of S2 the observed proportion is significantly (*p* = 0.007) higher as compared to the proportion of all neurons, while no significant (*p* = 0.170) difference was found in L5 of M1 (Figure 4A). In L6 we found marginally higher observed proportion of backlabeled cells in both M1 and S2, however, these differences were not significant. In the SP of M1 and S2, the observed proportion of backlabeled cells were higher than the proportions of all neurons, but the difference was significant (*p* = 0.001) only in the SP of M1 (Figure 4A).

In the M1 injected samples the observed proportion of backlabeled cells in the CP of S1Bf was significantly (*p* = 0.006) lower than the proportion of all neuron (Figure 4B). In contrast, a significantly (*p* = 0.007) higher proportion of backlabeled cells was observed in L5 of S1Bf (Figure 4B). In L6 the proportion of backlabeled cells was comparable (*p* = 0.176) and in the SP the proportion of backlabeled SP cells was virtually identical with the proportion of all neurons (Figure 4B).

For the S2 injected samples cell the proportion of backlabeled CP neurons in S1Bf was significantly (*p* = 0.001) lower than the proportion of all neurons (Figure 4B). In contrast, in L5 of S1Bf a significantly (*p* = 0.001) higher proportion of backlabeled was observed (Figure 4B). While in L6, the proportion of backlabeled cells was not significantly (*p* = 0.968) different from the proportion of all neurons, a significantly (*p* = 0.001) higher proportion of backlabeled SP cells was observed (Figure 4B).

In summary, these data indicate that the proportion of neurons projections from the CP toward the SP is consistently less than expected from the pure number of neurons, suggesting a low density of axonal projection from these neurons to adjacent neocortical areas. In contrast, for most investigated areas the number of projections from L5 toward the SP are higher than expected, indicating that these neurons already project to adjacent areas at P0. For SP we observed significantly more backlabeled cell in areas medial to the injection site, while the proportion of backlabeled SP cells lateral to the injection site was as expected from the number of SP cells (Figure 4B).

### Identification of functional synaptic connections between S1Bf, S2, and M1 in the P0 cortex

**FIGURE 5:**
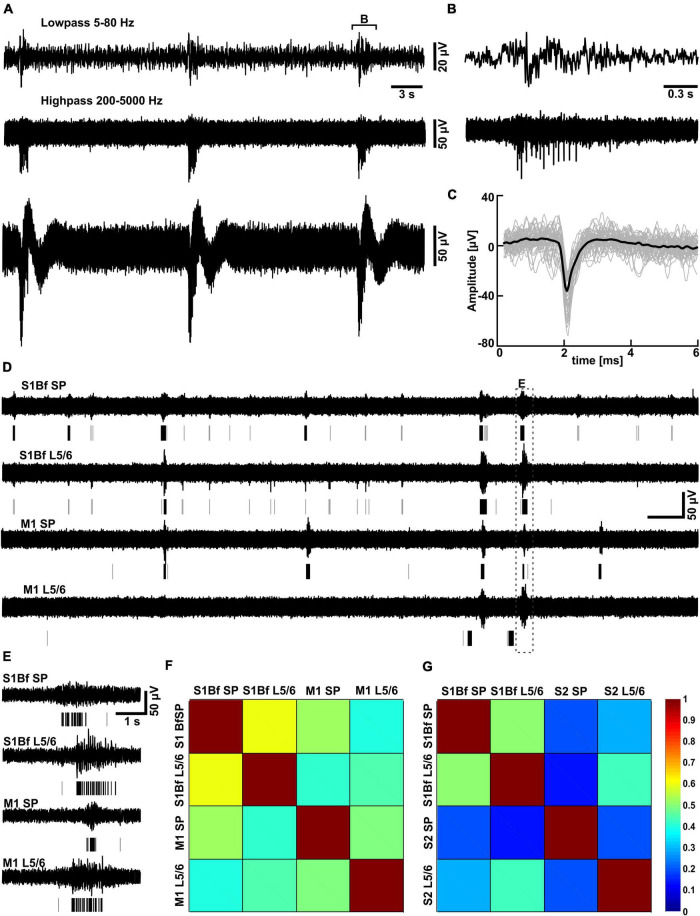
Partially correlated discontinuous multiunit activity in low Mg^2+^-ACSF. **(A)** Typical trace recorded with one MEA electrode located in L5/6 of S1Bf. Above the trace the highpass-filtered signal used for the spike detection and the lowpass-filtered signal are displayed. **(B)** Representative interval of a burst as depicted in panel **(A)**. **(C)** Plot of individual spikes identified by the threshold-crossing detector (gray traces) and the average spike waveform (black trace). **(D)** Simultaneous recording of multi-unit activity (MUA) in the subplate (SP) and in L5/6 of S1Bf and M1 recorded in low Mg^2+^ ACSF. The lines beneath the trace indicate identified spikes, isolated spikes are represented by gray lines, spikes belonging to a burst by black lines. Note the frequent occurrence of burst-like activity transients in all areas and that burst like activity was only partially correlated between the investigated regions. **(E)** Representative interval of a burst correlated between all electrodes [as indicated in panel **(A)**] illustrating a significant delay in the onsets of the bursts between the recordings. **(F)** The correlation matrix for electrode pairs inserted in S1Bf and M1 revealed that the bursts were only partially correlated between regions, with the highest degree of correlation observed between S1Bf SP and S1 L5/6 and the lowest degree of correlation between S1Bf SP and M1 L5/6. **(G)** Correlation matrix for electrode pairs inserted in S1Bf and S2. Note the overall lower degree of correlation between S1Bf and S2 as compared to S1Bf–M1 recordings.

In order to investigate whether the observed axonal projections are representing functional synaptic connections, we performed electrophysiological recordings using multishank electrode arrays positioned in visually identified regions of cortical slices, including M1, S1Bf, and S2 (Supplementary Figure 3). Because virtually no spiking activity could be observed in standard ACSF, we evoked spontaneous synchronous activity by bath application of the K^+^ channel blocker 4-AP in Mg^2+^-free ACSF (Richter et al., 2010). Under this condition the lack of Mg^2+^ unblocks NMDA receptors (Nowak et al., 1984) and reduces surface charge screening (Hahin and Campbell, 1983), while the unspecific K^+^ channel blocker 4-AP enhances the excitability (Perreault and Avoli, 1989). This condition evoked stable, burst like neuronal activity throughout the 1–1.5 h long recording sessions, which enables the identification of functional synaptic connectivity (Figures 5A–D). As this neuronal activity does not resemble the spindle-like burst typical for spontaneous network events in the immature cortex (Minlebaev et al., 2007; Yang et al., 2016), we analyzed only the MUA. MUA was identified in 200–5,000 Hz filtered traces using a simple threshold-crossing detector set at 5 times the standard deviation of a spike free interval (Figures 5A–C). It consisted of burst-like spike trains and few separated spikes (Figures 5D, E).

As immature cortical neurons show longer membrane time constants (McCormick and Prince, 1987; Luhmann et al., 2000), slower rising and falling phases of action potentials (Kriegstein et al., 1987), and a small number of synapses with low temporal fidelity (Hanganu et al., 2001; Doischer et al., 2008), we did not expect that the spikes of the immature neocortical network are synchronized with a millisecond precision. Indeed, 5–15 ms delay of monosynaptic connections in the newborn rodent neocortex has been previously demonstrated (Hanganu et al., 2002; Zhao et al., 2009; Myakhar et al., 2011). From these reports we assumed that a 5–15 ms temporal coincidence between spikes can represent a monosynaptic transmission. Therefore, we first generated cross-correlograms (CCGs) calculated for all pairs of electrodes located unequivocally in the SP and L5/6 (from *n* = 6 experiments for S1Bf-M1, *n* = 5 for S1-S2 and *n* = 5 for M1-S2 connections) and checked in this time window of 5–15 ms whether we could detect spike bins that are significantly above the threshold derived from surrogate data (see Section “Materials and methods”).

We observed above-threshold spike bins in some of the CCGs, with the occurrence varying between 15.8 and 53.3% of the pair-wise CCG from single experiments. However, the distribution of the above-threshold spikes did not show a typical clustering within the expected range of delay (Miller, 2000), but distributed over the whole analysis interval (data not shown). This result indicates that coincident spiking was not evident from the CCG analysis, suggesting that synaptic connections in the newborn cortex are too weak to establish a tight temporal spike correlation between single neurons. In addition, we cannot exclude that the immature properties of the neuronal excitability and the synaptic processes (Kriegstein et al., 1987; Luhmann et al., 2000; Hanganu et al., 2001; Doischer et al., 2008), also obscured the identification of a significant cross correlation between synaptically connected neurons.

To further characterize the functional connectivity between the different regions and layers we next investigated the temporal relation of burst firing.

### Coincident bursting analysis

All investigated areas showed consistent bursting activity under the recording conditions in 4-AP containing Mg^2+^-free ACSF (Figures 5A, D). Due to the shank distance we could only discriminate activity in the SP and in L5/6. The average occurrence of these bursts was between 2.0 ± 0.48 min^–1^ (*n* = 2,328 bursts in *n* = 11 slices) in the S1Bf SP and 1.1 ± 0.2 min^–1^ (*n* = 523 bursts in *n* = 6 slices) in M1 L5/6 (Table 3). The occurrence was significantly higher in the SP than in L5/6 for M1 (*p* = 0.046, *n* = 6 slices) and showed a strong tendency in S1 (*p* = 0.05, *n* = 11 slices). No significant differences between the areas were observed. The duration of the bursts was between 484 ± 67 ms in the S2 SP and 871 ± 107 ms in the S1Bf L5/6 (Table 3). The duration of the bursts in S1Bf L5/6 was significantly (*p* < 0.05) longer than in S1Bf SP, M1 L5/6, and SP and L5/6 of S2. In accordance with this longer burst duration, the number of spikes per burst were in S1Bf L5/6 (30 ± 5) significantly (*p* < 0.5) higher than in the other regions and layers (between 8 ± 2 and 18 ± 5) (Table 3).

**TABLE 3:** Properties of the burst discharges induced by the low Mg^2+^ solution at the different recording sites.

	Occurrence (min^–1^)	Duration (s)	Number of Spikes	Freq. (Hz)
S1 SP	2.04 ± 0.48 (*n* = 11)	604.8 ± 61.0 (*n* = 11)	10.0 ± 1.4 (*n* = 11)	19.4 ± 2.7 (*n* = 11)
S1 L5/6	1.16 ± 0.18 (*n* = 11)	871.3 ± 107.0 (*n* = 11)	29.5 ± 5.2 (*n* = 11)	28.2 ± 2.6 (*n* = 11)
M1 SP	1.92 ± 0.53 (*n* = 6)	830.3 ± 76.9 (*n* = 6)	13.5 ± 3.1 (*n* = 6)	15.6 ± 2.1 (*n* = 6)
M1 L5/6	1.06 ± 0.20 (*n* = 6)	791.4 ± 135.0 (*n* = 6)	14.6 ± 3.4 (*n* = 6)	20.3 ± 3.2 (*n* = 6)
S2 SP	1.97 ± 0.81 (*n* = 5)	484.1 ± 68.8 (*n* = 5)	7.5 ± 1.7 (*n* = 5)	16.9 ± 2.3 (*n* = 5)
S2 L5/6	0.91 ± 0.13 (*n* = 5)	664.2 ± 93.0 (*n* = 5)	18.0 ± 4.8 (*n* = 5)	24.9 ± 3.4 (*n* = 5)

Data represent mean ± S.E.M. The number of data points (*n*) refers to the number of slices investigated.

**FIGURE 6:**
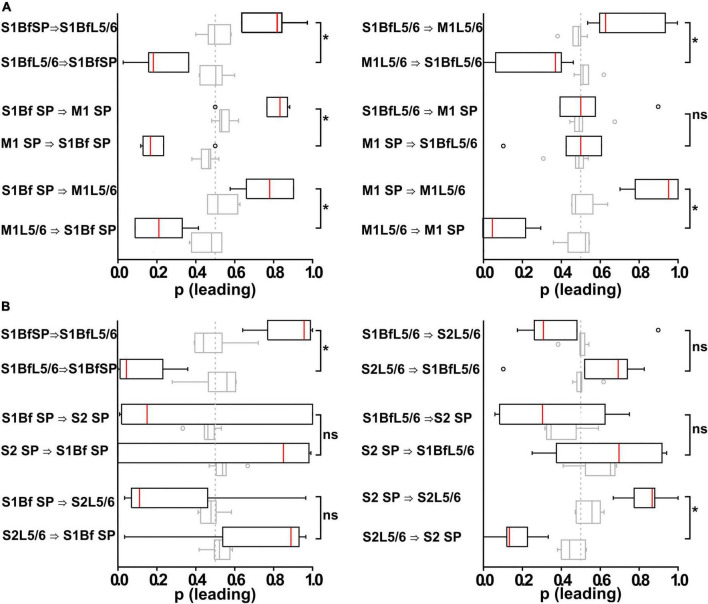
Analysis of burst onset differences in bursting multi-unit activity (MUA) activity between and within different layers in S1Bf, S2, and M1. **(A)** Box plots representing the relative occurrence (as normalized to the total number of correlated burst within an electrode pair) of burst onset at a given electrode pair inserted in S1BF and M1. Open circles represent identified outliers, shaded box plots represent the results of surrogate data sets and the dashed gray line at 0.5 represents a random distribution of burst onsets. Significant differences of burst onset between electrode pairs are indicated. Note the high occurrence of spike onsets in S1Bf SP, suggesting that neurons from the SP projecting to other areas might have a higher relevance for initiating postsynaptic local network activity. **(B)** As panel **(A)** but for an electrode pair using an electrode pair inserted in S1BF and S2. Note the absence of a significantly directionality except for the SP to Layer 5/6 connections within both regions. *p < 0.05; ns, no significant difference.

Intriguingly, the burst-like activity was not persistently synchronized across the investigated cortical areas (Choi et al., 2010), but non-stationary mutual synchronizations between distinct areas were found (see Figures 5D, E). For all recorded areas and layers, the ratio of synchronized bursts was significantly (*p* = 0.028) lower than one (which would correspond to a complete synchronization), but clearly above the synchronization ratios (between 0.007 and 0.028) calculated from surrogate datasets (Figures 5F, G and Tables 4, 5). We conclude from these observations that the functional connectivity in early postnatal cortical circuits is too weak to maintain stable synchronization, but that synaptic connections between the mutually synchronized areas must exist and can be used to estimate the functional connectivity between these areas. In order to estimate the favored direction of information flow between the investigated areas, we analyzed the latencies between the onsets of coincident bursts (Figure 6).

**TABLE 4:** Probability of coincident bursting estimated by S1Bf–M1 silicon probe pairs.

	S1 SP	S1 L5/6	M1 SP	M1 L5/6
S1 SP	1 ± 0.0 (*n* = 1,112)	0.59 ± 0.23 (*n* = 510)	0.52 ± 0.17 (*n* = 580)	0.39 ± 0.24 (*n* = 261)
S1 L5/6	0.59 ± 0.23 (*n* = 510)	1 ± 0.0 (*n* = 725)	0.41 ± 0.25 (*n* = 368)	0.44 ± 0.25 (*n* = 243)
M1 SP	0.52 ± 0.17 (*n* = 580)	0.41 ± 0.25 (*n* = 368)	1 ± 0.0 (*n* = 984)	0.50 ± 0.20 (*n* = 397)
M1 L5/6	0.39 ± 0.24 (*n* = 261)	0.44 ± 0.25 (*n* = 243)	0.50 ± 0.20 (*n* = 397)	1 ± 0.0 (*n* = 523)

The number of data points (*n*) refers to the number of coincident bursts detected in a total of six slices.

**TABLE 5:** Probability of coincident bursting estimated by S1Bf–S2 silicon probe pairs.

	S2 SP	S2 L5/6	S1 SP	S1 L5/6
S2 SP	1 ± 0.0 (*n* = 1,214)	0.16 ± 0.12 (*n* = 125)	0.21 ± 0.23 (*n* = 211)	0.09 ± 0.11 (*n* = 55)
S2 L5/6	0.16 ± 0.12 (*n* = 125)	1 ± 0.0 (*n* = 763)	0.27 ± 0.10 (*n* = 300)	0.43 ± 0.25 (*n* = 185)
S1 SP	0.21 ± 0.23 (*n* = 211)	0.27 ± 0.10 (*n* = 300)	1 ± 0.0 (*n* = 1,157)	0.38 ± 0.22 (*n* = 252)
S1 L5/6	0.09 ± 0.11 (*n* = 55)	0.43 ± 0.25 (*n* = 185)	0.38 ± 0.22 (*n* = 252)	1 ± 0.0 (*n* = 488)

The number of data points (*n*) refers to the number of coincident bursts detected in a total of five slices.

With this analysis we observed that in S1Bf, S2, and M1 the correlated bursts start significantly (*p* < 0.05) more often in the SP than in L5/6 (Figures 6A, B and Tables 6, 7), suggesting that synaptic connections from the SP to L5/6 contributed more reliably to the synchronization than the reciprocal synapses. In addition, the bursts started in S1Bf SP and L5/6 significantly (*p* > 0.05) more often than in the corresponding layers of M1 (Figure 6A and Table 6), whereas no significant onset preference was observed between S1Bf and S2 (Figure 6B). In summary, these significant differences in the latencies supports the concept of a reliable information flow from the SP to cortical layers in the early postnatal cortical network (Kanold and Luhmann, 2010; Viswanathan et al., 2017) and from S1 to M1 (Khazipov et al., 2004; An et al., 2014; Dooley and Blumberg, 2018; Tiong et al., 2019). In contrast, the low rates of correlated burst between S1 and S2 and the lack of a directed burst onset indicate that a functional coupling between S1 and S2 is only weakly developed in the early postnatal cortex.

**TABLE 6:** Directionality of connections between by S1Bf–M1 silicon probe pairs.

	Target region			
**Signal origin**	**S1 SP**	**S1 L5/6**	**M1 SP**	**M1 L5/6**
S1 SP		0.79 ± 0.12 (*n* = 510)	0.78 ± 0.13 (*n* = 580)	0.77 ± 0.12 (*n* = 261)
S1 L5/6	0.21 ± 0.12 (*n* = 510)		0.54 ± 0.18 (*n* = 368)	0.72 ± 0.18 (*n* = 243)
M1 SP	0.22 ± 0.13 (*n* = 580)	0.46 ± 0.18 (*n* = 368)		0.90 ± 0.12 (*n* = 397)
M1 L5/6	0.23 ± 0.12 (*n* = 261)	0.28 ± 0.18 (*n* = 243)	0.10 ± 0.12 (*n* = 397)	

The number of data points (*n*) refers to the number of coincident bursts analyzed in a total of six slices.

**TABLE 7:** Directionality of connections between by S1Bf–S2 silicon probe pairs.

	Target region			
**Signal origin**	**S2 SP**	**S2 L5/6**	**S1 SP**	**S1 L5/6**
S2 SP		0.86 ± 0.11 (*n* = 125)	0.77 ± 0.39 (*n* = 211)	0.73 ± 0.35 (*n* = 55)
S2 L5/6	0.14 ± 0.11 (*n* = 125)		0.65 ± 0.35 (*n* = 300)	0.55 ± 0.26 (*n* = 185)
S1 SP	0.23 ± 0.39 (*n* = 211)	0.35 ± 0.35 (*n* = 300)		0.78 ± 0.20 (*n* = 252)
S1 L5/6	0.27 ± 0.35 (*n* = 55)	0.45 ± 0.26 (*n* = 185)	0.22 ± 0.20 (*n* = 252)	

The number of data points (*n*) refers to the number of coincident bursts analyzed in a total of five slices.

## Discussion

Several studies demonstrated that the SP is not only a waiting station for ingrowing thalamocortical axons, but rather that anatomical and functional connections between SP neurons and overlying cortical layers exist, transmitting information from subcortical sources to the respective neocortical area (Friauf et al., 1990; Finney et al., 1998; Kanold et al., 2003; Kanold and Shatz, 2006; Hanganu et al., 2009; Zhao et al., 2009; Tolner et al., 2012; Viswanathan et al., 2017; Wess et al., 2017; Molnar et al., 2020; Meng et al., 2021). But to which extent the SP also contributes to the information flow between adjacent neocortical areas during early development has been addressed only by one study, which identified anatomical projections from S1 to M1 at P2 (Tiong et al., 2019). In the present study we therefore systematically investigated the SP directed lateral connectivity between S1, S2 and M1 in the P0 mouse brain and performed electrophysiological experiments to analyze whether reliable functional synaptic connections between these areas exists at this early postnatal stage.

### Anatomical corticocortical network in newborn mouse

We identified neurons that project toward the SP by prominent DiI backlabeling in their somata. These analyses demonstrated that already at P0 a substantial number of backlabeled neurons can be identified in adjacent functional areas. The low number of backlabeled neurons and the high inter-individual variance in these numbers are probably due to the required use of tiny DiI crystals in the present study. For the interpretation of our results one must keep in mind that the carbocyanate-dye DiI labels all projections that traverse the area in which the small DiI crystal was placed. The remote backlabeled cell bodies observed upon DiI injection at the dense dendritic arbor of the SP cells (SP-L6 border) in M1, S1Bf, and S2 therefore represent mainly neurons that send long-range projections through this injection site (Hoerder-Suabedissen and Molnar, 2012), whereas we cannot predict from these experiments whether they terminate in the SP and/or synapse on SP neurons.

Projections from S1Bf toward the SP in M1 and S2, as well as projections from M1 or S2 toward the SP of S1Bf, were observed. This result demonstrates that neocortical neurons do not only project toward the SP within the same functional column (Kostović and Rakic, 1980; Hanganu et al., 2002; Hirsch and Luhmann, 2008; Viswanathan et al., 2012), but also project toward adjacent functional areas. The existence of a SP projection between S1 and M1 has already been demonstrated in the P2 murine neocortex (Tiong et al., 2019), however, the present results advanced the appearance of this projection to P0. In contrast, no projections between M1 and S2 could be found in our specimens, suggesting a lack of connectivity between these more distant areas. It was previously described in the P0 mouse neocortex, that neurons from different layers of the dorso-medial cortex send cortico-fugal axons through the internal capsule (Auladell et al., 2000). In our samples, S1Bf backlabeled cells after S2 injections may thus partially represent such projections. The presence of early cortico-spinal projections in the motor cortex has also been described previously (Gu et al., 2017), therefore the M1 backlabeled cells in S1Bf injected samples might also represent such projections. In contrast, the contribution of cortico-fugal projections in S2 after S1Bf injections to S2 backlabeled cells can be excluded, because S2 cortico-thalamic fibers do not cross the SP of S1Bf (Grant et al., 2012). We can also exclude, that S1Bf backlabeled cells on S2 injected samples project *via* the SP region toward insular cortices, as these projections are not present earlier than P3-4 (Sohur et al., 2014). Thus the latter projections may represent specific connections from S2 toward the SP of S1Bf.

**FIGURE 7:**
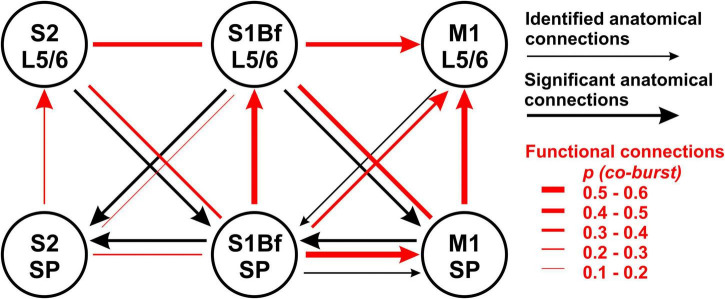
Summary diagram for comparison of anatomical projections and functional connectivity between M1, S1Bf, and S2. Black arrows indicate the direction of backlabeled cell axonal projections toward the SP. Thick black arrows indicate if backlabeled cell number was significantly higher than expected. The red lines depict the estimated connectivity between two regions, as determined by the burst correlation. Arrowheads indicate a significant direction of burst correlation, as determined from burst initiation latencies. Note the clear direction in the functional connectivity from SP to L5/6 in all investigated regions and from S1Bf to M1.

In addition, we observed an obvious difference in the proportion of cells from different layers that projects toward the SP. The fraction of projections is higher than expected from the proportion of neurons for the projections from L5/6 toward the SP between S2 and S1Bf, between S1Bf and S2, as well as between S1Bf and M1. In contrast, less L5/6 to SP connections were found between M1 and S1B. For SP we observed significantly more backlabeled cells in areas medial to the injection site, while the frequency of backlabeled SP cells lateral to the injection site was as expected from the number of SP cells (Figure 7 and Table 2). The latter observation suggests directed axonal projections from S1 toward S2 and from M1 toward S1 on the level of SP-SP connections, although we cannot exclude that this asymmetrical connectivity within the SP may reflect a general trend favoring mediolateral axonal projections. Finally, the frequency of projections from CP neurons toward the SP is consistently less than expected for all identified connections, in line with the immature morphological properties of CP neurons (Kasper et al., 1994).

A striking observation in our study is that we failed to identify any backlabeled cell in M1 upon DiI injection in S2 as well as backlabeled cells in S2 upon DiI injection in M1. This observation suggests that in the P0 neocortex neurons project preferably to cortical regions in the closest proximity, while connections to more distant areas are not established yet. In the adult neocortex functional synaptic connections between S2 and M1 exists and are relevant for motosensory integration (Suter and Shepherd, 2015). We can exclude that the lack of backlabeled neurons in these more distant regions were caused by an insufficient DiI diffusion within the 4 week incubation period. In accordance with previous results (Schlaggar and O’Leary, 1994; Agmon et al., 1995) we were able to identify in our probes backlabeled cells in the thalamus upon DiI injection in S1Bf or S2 (data not shown), indicating that DiI can reliably label neurons at an estimated axonal distance of >3,500 μm (manual measurement along S1Bf thalamocortical axons in Fiji; Schindelin et al., 2012). Thus we assume that the lack of backlabeled cells in distant cortical regions (<2,500 μm apart), does not represent insufficient fluorophore dispersion, but a lack of connectivity between both regions in the P0 neocortex. On the other hand, all connections that project above the sliced surface were cut in our preparation, thus we cannot exclude that the longer connections between S2 and M1 have a higher probability to be removed.

### Functional synaptic connections between M1, S1Bf, and S2

In line with previous studies (Golshani and Jones, 1999; Abdelmalik et al., 2005; Dupont et al., 2006), substantial neuronal activity can be observed in the immature cortex *in vitro* only under an excitatory regime, in our case upon addition of the K^+^ channel blocker 4-AP in Mg^2+^-free ACSF. A substantial portion of the induced bursting activity consists only of local activity that did not propagate to adjacent cortical areas or layers. The observation that in the P0 neocortex the bursting activity was not consistently synchronized between adjacent layers and/or functional areas indicates that the synaptic connectivity was not as strong as in the more mature cortex during later postnatal stages (Bureau et al., 2004; Doischer et al., 2008; Choi et al., 2010; An et al., 2014). Our results are in line with previous observations demonstrating that in the developing neocortex spontaneous network activity is restricted to functional areas or even functional columns (Garaschuk et al., 2000; Khazipov and Luhmann, 2006; Yang et al., 2013; Luhmann et al., 2016; Luhmann and Khazipov, 2018; Mizuno et al., 2018; Pires et al., 2021).

However, between 39 and 52% of the bursting activity is temporally correlated in different layers in S1Bf and M1 (Table 4). This fraction was lower in S1Bf and S2 (9–43%, Table 5). For several of the investigated areas/layers we found that the sequence of bursting activity showed a significant directionality (see Table 6 and Figure 7). We assume that this significant sequence of burst initiation reflects a dominance of synaptic connections from the area in which the bursting activity appears first to the area with the subsequent burst. This assumption suggests that the amount of synaptic contacts is not strong enough to reliably trigger bursting activity in the postsynaptic area, but that the amount of synaptic inputs can be sufficient to significantly increase the probability to induce a local burst in the postsynaptic area. Thus we suppose that the identification of a significant directionality in the burst initiation between two areas indicates that the functional synaptic connectivity in this preferred direction is larger than the reciprocal connections. Figure 7 summarizes the analysis of the differences in the burst onset. An obvious directed functional connectivity, as illustrated by the bold red arrows, was observed from the SP toward L5/6 of both S1Bf and M1 as well as from S1Bf toward the M1 in both SP and L5/6. A weaker functional directed connectivity appeared between S2 SP and S2 L5/6 and from S1Bf SP toward M1 L5/6.

Two major conclusions can be drawn from these findings. First, we observed for all regions a significant synaptic directionality from the SP to L5/6 of the same area. For S1Bf this observation is in line with all information that is known about the implication of SP cells in the formation of early synaptic circuits in various primary sensory areas, mediating transfer of thalamic information toward the overlying cortical layers in sensory cortical areas (Friauf et al., 1990; Finney et al., 1998; Hanganu et al., 2009; Zhao et al., 2009; Myakhar et al., 2011; Viswanathan et al., 2012). However, our results also indicate that the SP may play a role in establishing the transient synaptic circuit mediating transfer of information from subcortical areas to upper layers in M1 and, probably to a lower extend, in S2.

Second we observed a significant directionality from both S1BF SP and S1Bf L5/6 toward the corresponding regions of M1, suggesting that the synaptic connections from S1Bf toward M1 are stronger than the reciprocal connections. For sensory evoked activity in the P3 mouse cortex it has been shown that S1 activity precedes M1 activity (Khazipov et al., 2004; An et al., 2014; Dooley and Blumberg, 2018). While we cannot exclude that the organization of thalamocortical inputs contribute to the delayed M1 activity, the early synaptic connectivity between S1 and M1 suggested by our results may pave the information flow between these areas in the early postnatal cortex. In contrast, between S1Bf and S2 only a very weak temporal correlation between the bursts and no significant directionality of burst onsets were observed. This finding suggests that only a weak synaptic connection exists between S1 and S2 in the early postnatal brain, which is in line with a recent *in vivo* study demonstrating that whisker-related activity in the S2 regions is smaller and that a consistent activity representing a solid interaction between both areas establishes only around P6 (Cai et al., 2022). Importantly, the significant information transfer from S1Bf to M1 occurred both between L5/6 and between the SP, while the connection from S1Bf SP to M1 L5/6 is less prominent (Figure 7 and Table 4). This observation suggests that in the P0 neocortex not only L5/6 neurons are functionally coupled, but that probably also direct synaptic connections between SP neurons of S1Bf and M1 exist.

The assumed strength of the functional synaptic connections did not fit in all instances to proportions of axonal connections estimated from the DiI tracing (Figure 7). Possible explanations for these contradictory results are as follows: (i) the observation of backlabeled cells upon DiI injection in the target region does not necessarily represent a synaptic connection between the labeled cell and neurons in the target region, (ii) the statistical analysis of the distribution of the backlabeled cells across cortical layers reveals exclusively a deviation from the cell numbers expected from the total neuron number, which necessarily overestimates the number of structurally and functionally mature neurons in the heterogeneous populations at such early developmental stages, (iii) SP cells may receive connections *via* silent synapses (Meng et al., 2014; Kanold et al., 2019) thereby causing a deviation between structural and functional connectivity, and (iv) SP and upper layer cortical cell neurites undergo synaptic pruning (Hua and Smith, 2004; Hoerder-Suabedissen and Molnar, 2015), proposing that the axons of backlabeled cells crossing these regions are not necessarily functional.

### The SP mediates synaptic interactions between M1, S1Bf, and S2 already at P0

In the adult cerebral cortex connections between M1, S1Bf, and S2 play a crucial role in sensorimotor integration (Sreenivasan et al., 2016; Hubatz et al., 2020). The formation of specific synaptic connections between those functionally interconnected areas depends not only on genetic programs, but also on the patterns of spontaneous and sensory evoked activity during fetal and postnatal development (Barkat et al., 2011; Erzurumlu and Gaspar, 2012; Levelt and Hubener, 2012; Luhmann et al., 2016, but see Molnár et al., 2002). The connections described in the present study provide an anatomical template that can serve to transmit or establish local and global correlated activity (Garaschuk et al., 2000; Blankenship and Feller, 2010; Leighton and Lohmann, 2016; Luhmann et al., 2016; Luhmann and Khazipov, 2018).

A variety of anatomical and functional studies demonstrated that for the establishment of functional connectivity in primary sensory cortices transient excitatory connections from the SP toward superficially located cortical layers are essential (Ghosh et al., 1990; Kanold et al., 2003; Kanold and Shatz, 2006; Tolner et al., 2012; Molnar et al., 2020; Meng et al., 2021). The functional axonal projections between SP and L5/6 in S1Bf identified in the preset study support these previous findings. However, no details about the ontogeny of synaptic projections from the SP toward superficial cortical layers have been published for M1 and secondary sensory cortices. Our study demonstrates that in M1 and S2 synaptic connections from SP neurons toward upper cortical layers can be also an essential functional element that serve to establish a transient network required for the adequate development of the circuits. In particular for M1 the role of the functional transient connectivity awaits further investigation, as it has been shown that the SP of the rodent motor cortex is thicker than in sensory areas (Qu et al., 2016) and motor deficits have been described after hypoxia-induced lesion within the subplate (McQuillen et al., 2003). Accordingly, it has been proposed that the SP of M1 plays an important role for the development of motor functions (Hadders-Algra, 2007).

In addition to these vertical connections within a cortical column, transient correlated activity between functionally related areas also plays an essential role for the acquisition of the interconnectivity between these areas (Khazipov and Luhmann, 2006; Yang et al., 2013; Sieben et al., 2015; Luhmann et al., 2016; Mezzera and Lopez-Bendito, 2016; Luhmann and Khazipov, 2018; Xu et al., 2020). The adult M1 processes somatosensory information and thus receives thalamic input (Hooks et al., 2013), but a major part of sensory inputs originate from S1 (Farkas et al., 1999). Thus an intense interconnection between these areas must be generated during early development. Our data demonstrate that on the level of the SP a reciprocal axonal projection between M1 and S1Bf exists already in the P0 mouse neocortex, suggesting that the SP can integrate neuronal activity of these functionally related areas already during the earliest stages. A variety of studies suggested that M1 is involved in somatosensory information processing already in the immature neocortex. During early developmental stages the majority of spontaneous movements are initiated in subcortical areas and transmitted *via* sensory feedback to M1 (Khazipov et al., 2004; Tiriac et al., 2014; Dooley and Blumberg, 2018). Already in P1 to P3 rats sensory stimulation induces a reliably activation of M1 (Khazipov et al., 2004; An et al., 2014). The observation that in the early postnatal neocortex (P1-P3) part of the correlated activity in S1 and M1 is at this age not associated with movements (Khazipov et al., 2004; An et al., 2014), indicates that a direct link between S1 and M1 must exist already at this age. While our data indicate that part of this activation can be mediated by synaptic connections in L5/6, the S1Bf to M1 connections within the SP can also contribute to this information transfer. In contrast to this connection between S1Bf and M1, our experimental results indicate that the functional connectivity between S1 and S2 is less developed at P0, although we observed in the SP anatomical projections between S2 and S1Bf. Correlated neuronal activity occurs in this region at P6-8 (Cai et al., 2022), suggesting that the connectivity toward secondary sensory areas develops delayed, as has been also shown for associative cortical areas (Sydnor et al., 2021).

The cortex of a newborn mouse can roughly be compared to the developmental state of the human neocortex in the 18th gestational week (Workman et al., 2013), a stage when the thick human subplate is established (Kostović, 2020). In the human cortex the subplate zone probably plays a more prominent role than in rodents, as judged by the sheer thickness and complexity of this structure (Kostović and Rakić, 1990; Duque et al., 2016). The early appearance of oblique, horizontally oriented axons in the human subplate indicate that it not only is a waiting station for thalamocortical axons (Allendoerfer and Shatz, 1994), but also contains corticocortical axons (Duque et al., 2016). Thus the subplate may serve as an interface for both thalamic and corticocortical connections already during early fetal developmental stages (Kostović and Rakić, 1990). The identification of lateral connectivity within the SP provided by the present study supports this hypothesis. Thereby the subplate can contribute to the transient occurrence of widespread synchronized electroencephalogram (EEG) activity in the immature human brain (Wallois et al., 2021) and control the establishment of adequate synaptic connections between functionally related cortical areas.

## Data availability statement

The raw data supporting the conclusions of this article will be made available by the authors, without undue reservation.

## Ethics statement

This animal study was reviewed and approved by the Landesuntersuchungsamt Rheinland-Pfalz, Koblenz, Germany.

## Author contributions

LG: conceptualization, investigation, methodology, writing—original draft, sample preparations, immunostainings, data acquisition, image and data analysis, acute slice preparations, electrophysiological recordings, and data analysis. WK: data analysis, critical revision, and review and editing. HL: conceptualization, review and editing, critical revision, supervision, and funding acquisition. All authors contributed to the article and approved the submitted version.

## Supplementary material

Supplementary Figure 1 Representative photomicrographs of DiI crystal injections at the SP-layer 6 border of M1 **(A)**, S1Bf **(B)**, and S2 **(C)** in coronal sections of P0 mouse brains. Immediately after DiI injection, photomicrographs were captured with an adequate fluorescence filter set for SYTOX™ Green nuclear marker and for DiI. White lines demark the DiI injection target areas from the adjacent sensory areas as estimated from reference points in the developing mouse brain atlas (Paxinos, 2007). Scale bar is 1 mm.

Supplementary Figure 2 Scheme for assigning the cortical layer boundaries and for determination of neuron number proportions across cortical layer to estimate expected backlabeled cell frequencies. Scale bar is 100 μm.

Supplementary Figure 3 Composite image of the DiI signal and the pseudocoloured DAPI signal showing silicon probe positioning in the M1 S1Bf panel **(A)** and S1Bf S2 panel **(B)** configuration. Position of the analyzed L5/6 and SP shanks is marked with red circles. Scale bar is 500 μm.

## References

[B1] Abdelmalik P. A. McIntyre Burnham W. Carlen P. L. (2005). Increased seizure susceptibility of the hippocampus compared with the neocortex of the immature mouse brain in vitro. *Epilepsia* 46 356–366. 10.1111/j.0013-9580.2005.34204.x 15730532

[B2] Ackman J. B. Burbridge T. J. Crair M. C. (2012). Retinal waves coordinate patterned activity throughout the developing visual system. *Nature* 490 219–225. 10.1038/nature11529 23060192 PMC3962269

[B3] Agmon A. Yang L. T. Jones E. G. O’Dowd D. K. (1995). Topological precision in the thalamic projection to neonatal mouse barrel cortex. *J. Neurosci.* 15 549–561. 10.1523/JNEUROSCI.15-01-00549.1995 7823163 PMC6578331

[B4] Allendoerfer K. L. Shatz C. J. (1994). The subplate, a transient neocortical structure: Its role in the development of connections between thalamus and cortex. *Annu. Rev. Neurosci.* 17 185–218. 10.1146/annurev.ne.17.030194.001153 8210173

[B5] An S. Kilb W. Luhmann H. J. (2014). Sensory-evoked and spontaneous gamma and spindle bursts in neonatal rat motor cortex. *J. Neurosci.* 34 10870–10883. 10.1523/JNEUROSCI.4539-13.2014 25122889 PMC6705262

[B6] Anton-Bolanos N. Sempere-Ferrandez A. Guillamon-Vivancos T. Martini F. J. Perez-Saiz L. Gezelius H. (2019). Prenatal activity from thalamic neurons governs the emergence of functional cortical maps in mice. *Science* 364 987–990. 10.1126/science.aav7617 31048552 PMC7611000

[B7] Arganda-Carreras I. Kaynig V. Rueden C. Eliceiri K. W. Schindelin J. Cardona A. (2017). Trainable Weka Segmentation: A machine learning tool for microscopy pixel classification. *Bioinformatics* 33 2424–2426. 10.1093/bioinformatics/btx180 28369169

[B8] Auladell C. Perez-Sust P. Super H. Soriano E. (2000). The early development of thalamocortical and corticothalamic projections in the mouse. *Anat. Embryol. (Berl.)* 201 169–179. 10.1007/PL00008238 10664178

[B9] Barkat T. R. Polley D. B. Hensch T. K. (2011). A critical period for auditory thalamocortical connectivity. *Nat. Neurosci.* 14 1189–1194. 10.1038/nn.2882 21804538 PMC3419581

[B10] Blankenship A. G. Feller M. B. (2010). Mechanisms underlying spontaneous patterned activity in developing neural circuits. *Nat. Rev. Neurosci.* 11 18–29. 10.1038/nrn2759 19953103 PMC2902252

[B11] Boon J. Clarke E. Kessaris N. Goffinet A. Molnar Z. Hoerder-Suabedissen A. (2019). Long-range projections from sparse populations of GABAergic neurons in murine subplate. *J. Comp. Neurol.* 527 1610–1620. 10.1002/cne.24592 30520039 PMC6492162

[B12] Brecht M. (2007). Barrel cortex and whisker-mediated behaviors. *Curr. Opin. Neurobiol.* 17 408–416. 10.1016/j.conb.2007.07.008 17702566

[B13] Brockmann M. D. Poschel B. Cichon N. Hanganu-Opatz I. L. (2011). Coupled oscillations mediate directed interactions between prefrontal cortex and hippocampus of the neonatal rat. *Neuron* 71 332–347. 10.1016/j.neuron.2011.05.041 21791291

[B14] Bureau I. Shepherd G. M. Svoboda K. (2004). Precise development of functional and anatomical columns in the neocortex. *Neuron* 42 789–801. 10.1016/j.neuron.2004.05.002 15182718

[B15] Cai L. Yang J. W. Wang C. F. Chou S. J. Luhmann H. J. Karayannis T. (2022). Identification of a developmental switch in information transfer between whisker S1 and S2 cortex in mice. *J. Neurosci.* 42 4435–4448. 10.1523/JNEUROSCI.2246-21.2022 35501157 PMC9172289

[B16] Chen J. L. Carta S. Soldado-Magraner J. Schneider B. L. Helmchen F. (2013). Behaviour-dependent recruitment of long-range projection neurons in somatosensory cortex. *Nature* 499 336–340. 10.1038/nature12236 23792559

[B17] Choi J. H. Koch K. P. Poppendieck W. Lee M. Shin H. S. (2010). High resolution electroencephalography in freely moving mice. *J. Neurophysiol.* 104 1825–1834. 10.1152/jn.00188.2010 20610789

[B18] Del Rio-Bermudez C. Blumberg M. S. (2018). Active sleep promotes functional connectivity in developing sensorimotor networks. *Bioessays* 40:e1700234. 10.1002/bies.201700234 29508913 PMC6247910

[B19] Doischer D. Hosp J. A. Yanagawa Y. Obata K. Jonas P. Vida I. (2008). Postnatal differentiation of basket cells from slow to fast signaling devices. *J. Neurosci.* 28 12956–12968. 10.1523/JNEUROSCI.2890-08.2008 19036989 PMC6671784

[B20] Dooley J. C. Blumberg M. S. (2018). Developmental ‘awakening’ of primary motor cortex to the sensory consequences of movement. *Elife* 7:e41841. 10.7554/eLife.41841.024 30574868 PMC6320070

[B21] Dupont E. Hanganu I. L. Kilb W. Hirsch S. Luhmann H. J. (2006). Rapid developmental switch in the mechanisms driving early cortical columnar networks. *Nature* 439 79–83. 10.1038/nature04264 16327778

[B22] Duque A. Krsnik Z. Kostović I. Rakic P. (2016). Secondary expansion of the transient subplate zone in the developing cerebrum of human and nonhuman primates. *Proc. Natl. Acad. Sci. U.S.A.* 113 9892–9897. 10.1073/pnas.1610078113 27503885 PMC5024589

[B23] Erzurumlu R. S. Gaspar P. (2012). Development and critical period plasticity of the barrel cortex. *Eur. J. Neurosci.* 35 1540–1553. 10.1111/j.1460-9568.2012.08075.x 22607000 PMC3359866

[B24] Farkas T. Kis Z. Toldi J. Wolff J. R. (1999). Activation of the primary motor cortex by somatosensory stimulation in adult rats is mediated mainly by associational connections from the somatosensory cortex. *Neuroscience* 90 353–361. 10.1016/S0306-4522(98)00451-5 10215140

[B25] Fassihi A. Akrami A. Pulecchi F. Schonfelder V. Diamond M. E. (2017). Transformation of perception from sensory to motor cortex. *Curr. Biol.* 27 1585–1596. 10.1016/j.cub.2017.05.011 28552362 PMC5462624

[B26] Feldmeyer D. Brecht M. Helmchen F. Petersen C. C. Poulet J. F. Staiger J. F. (2013). Barrel cortex function. *Prog. Neurobiol.* 103 3–27. 10.1016/j.pneurobio.2012.11.002 23195880

[B27] Finney E. M. Stone J. R. Shatz C. J. (1998). Major glutamatergic projection from subplate into visual cortex during development. *J. Comp. Neurol.* 398 105–118. 10.1002/(SICI)1096-9861(19980817)398:1<105::AID-CNE7>3.0.CO;2-5 9703030

[B28] Friauf E. Mcconnell S. K. Shatz C. J. (1990). Functional synaptic circuits in the subplate during fetal and early postnatal development of cat visual cortex. *J. Neurosci.* 10 2601–2613. 10.1523/JNEUROSCI.10-08-02601.1990 2388080 PMC6570284

[B29] Gaïarsa J. L. Khalilov I. Gozlan H. Ben-Ari Y. (2001). Morphology of CA3 non-pyramidal cells in the developing rat hippocampus. *Brain Res. Dev. Brain Res.* 127 157–164. 10.1016/S0165-3806(01)00130-4 11335002

[B30] Garaschuk O. Linn J. Eilers J. Konnerth A. (2000). Large-scale oscillatory calcium waves in the immature cortex. *Nat. Neurosci.* 3 452–459. 10.1038/74823 10769384

[B31] Ghosh A. Antonini A. Mcconnell S. K. Shatz C. J. (1990). Requirement for subplate neurons in the formation of thalamocortical connections. *Nature* 347 179–181. 10.1038/347179a0 2395469

[B32] Golshani P. Jones E. G. (1999). Synchronized paroxysmal activity in the developing thalamocortical network mediated by corticothalamic projections and “silent” synapses. *J. Neurosci.* 19 2865–2875. 10.1523/JNEUROSCI.19-08-02865.1999 10191304 PMC6782276

[B33] Gomez L. J. Dooley J. C. Sokoloff G. Blumberg M. S. (2021). Parallel and serial sensory processing in developing primary somatosensory and motor cortex. *J. Neurosci.* 41 3418–3431. 10.1523/JNEUROSCI.2614-20.2021 33622773 PMC8051688

[B34] Grant E. Hoerder-Suabedissen A. Molnar Z. (2012). Development of the corticothalamic projections. *Front. Neurosci.* 6:53. 10.3389/fnins.2012.00053 22586359 PMC3343305

[B35] Gu Z. Kalambogias J. Yoshioka S. Han W. Li Z. Kawasawa Y. I. (2017). Control of species-dependent cortico-motoneuronal connections underlying manual dexterity. *Science* 357 400–404. 10.1126/science.aan3721 28751609 PMC5774341

[B36] Gul-Mohammed J. Arganda-Carreras I. Andrey P. Galy V. Boudier T. (2014). A generic classification-based method for segmentation of nuclei in 3D images of early embryos. *BMC Bioinformatics* 15:9. 10.1186/1471-2105-15-9 24423252 PMC3900670

[B37] Hadders-Algra M. (2007). Putative neural substrate of normal and abnormal general movements. *Neurosci. Biobehav. Rev.* 31 1181–1190. 10.1016/j.neubiorev.2007.04.009 17568672

[B38] Hahin R. Campbell D. T. (1983). Simple shifts in the voltage dependence of sodium channel gating caused by divalent cations. *J. Gen. Physiol.* 82 785–805. 10.1085/jgp.82.6.785 6319538 PMC2228720

[B39] Hanganu I. L. Ben-Ari Y. Khazipov R. (2006). Retinal waves trigger spindle bursts in the neonatal rat visual cortex. *J. Neurosci.* 26 6728–6736. 10.1523/JNEUROSCI.0752-06.2006 16793880 PMC6673818

[B40] Hanganu I. L. Kilb W. Luhmann H. J. (2001). Spontaneous synaptic activity of subplate neurons in neonatal rat somatosensory cortex. *Cereb. Cortex* 11 400–410. 10.1093/cercor/11.5.400 11313292

[B41] Hanganu I. L. Kilb W. Luhmann H. J. (2002). Functional synaptic projections onto subplate neurons in neonatal rat somatosensory cortex. *J. Neurosci.* 22 7165–7176. 10.1523/JNEUROSCI.22-16-07165.2002 12177212 PMC6757868

[B42] Hanganu I. L. Okabe A. Lessmann V. Luhmann H. J. (2009). Cellular mechanisms of subplate-driven and cholinergic input-dependent network activity in the neonatal rat somatosensory cortex. *Cereb. Cortex* 19 89–105. 10.1093/cercor/bhn061 18440948

[B43] Hirsch S. Luhmann H. J. (2008). Pathway-specificity in N-methyl-D-aspartate receptor-mediated synaptic inputs onto subplate neurons. *Neuroscience* 153 1092–1102. 10.1016/j.neuroscience.2008.01.068 18455878

[B44] Hoerder-Suabedissen A. Molnar Z. (2012). Morphology of mouse subplate cells with identified projection targets changes with age. *J. Comp. Neurol.* 520 174–185. 10.1002/cne.22725 21800308

[B45] Hoerder-Suabedissen A. Molnar Z. (2015). Development, evolution and pathology of neocortical subplate neurons. *Nat. Rev. Neurosci.* 16 133–146. 10.1038/nrn3915 25697157

[B46] Hooks B. M. Mao T. Gutnisky D. A. Yamawaki N. Svoboda K. Shepherd G. M. (2013). Organization of cortical and thalamic input to pyramidal neurons in mouse motor cortex. *J. Neurosci.* 33 748–760. 10.1523/JNEUROSCI.4338-12.2013 23303952 PMC3710148

[B47] Hua J. Y. Smith S. J. (2004). Neural activity and the dynamics of central nervous system development. *Nat. Neurosci.* 7 327–332. 10.1038/nn1218 15048120

[B48] Hubatz S. Hucher G. Shulz D. E. Ferezou I. (2020). Spatiotemporal properties of whisker-evoked tactile responses in the mouse secondary somatosensory cortex. *Sci. Rep.* 10:763. 10.1038/s41598-020-57684-6 31964984 PMC6972923

[B49] Kanold P. O. Luhmann H. J. (2010). The subplate and early cortical circuits. *Annu. Rev. Neurosci.* 33 23–48. 10.1146/annurev-neuro-060909-153244 20201645

[B50] Kanold P. O. Shatz C. J. (2006). Subplate neurons regulate maturation of cortical inhibition and outcome of ocular dominance plasticity. *Neuron* 51 627–638. 10.1016/j.neuron.2006.07.008 16950160

[B51] Kanold P. O. Deng R. Meng X. (2019). The integrative function of silent synapses on subplate neurons in cortical development and dysfunction. *Front. Neuroanat.* 13:41. 10.3389/fnana.2019.00041 31040772 PMC6476909

[B52] Kanold P. O. Kara P. Reid R. C. Shatz C. J. (2003). Role of subplate neurons in functional maturation of visual cortical columns. *Science* 301 521–525. 10.1126/science.1084152 12881571

[B53] Kasper E. M. Lubke J. Larkman A. U. Blakemore C. (1994). Pyramidal neurons in layer 5 of the rat visual cortex. III. Differential maturation of axon targeting, dendritic morphology, and electrophysiological properties. *J. Comp. Neurol.* 339 495–518. 10.1002/cne.903390404 8144743

[B54] Ke M. T. Fujimoto S. Imai T. (2013). SeeDB: A simple and morphology-preserving optical clearing agent for neuronal circuit reconstruction. *Nat. Neurosci.* 16 1154–1156. 10.1038/nn.3447 23792946

[B55] Khazipov R. Luhmann H. J. (2006). Early patterns of electrical activity in the developing cerebral cortex of humans and rodents. *Trends Neurosci.* 29 414–418. 10.1016/j.tins.2006.05.007 16713634

[B56] Khazipov R. Sirota A. Leinekugel X. Holmes G. L. Ben-Ari Y. Buzsaki G. (2004). Early motor activity drives spindle bursts in the developing somatosensory cortex. *Nature* 432 758–761. 10.1038/nature03132 15592414

[B57] Kostović I. (2020). The enigmatic fetal subplate compartment forms an early tangential cortical nexus and provides the framework for construction of cortical connectivity. *Prog. Neurobiol.* 194:101883. 10.1016/j.pneurobio.2020.101883 32659318

[B58] Kostović I. Rakic P. (1980). Cytology and time of origin of interstitial neurons in the white matter in infant and adult human and monkey telencephalon. *J. Neurocytol.* 9 219–242. 10.1007/BF01205159 7441294

[B59] Kostović I. Rakić P. (1990). Developmental history of the transient subplate zone in the visual and somatosensory cortex of the macaque monkey and human brain. *J. Comp. Neurol.* 297 441–470. 10.1002/cne.902970309 2398142

[B60] Kriegstein A. R. Suppes T. Prince D. A. (1987). Cellular and synaptic physiology and epileptogenesis of developing rat neocortical neurons in vitro. *Brain Res.* 431 161–171. 10.1016/0165-3806(87)90206-9 3040188

[B61] Leighton A. H. Lohmann C. (2016). The wiring of developing sensory circuits-from patterned spontaneous activity to synaptic plasticity mechanisms. *Front. Neural Circuits* 10:71. 10.3389/fncir.2016.00071 27656131 PMC5011135

[B62] Levelt C. N. Hubener M. (2012). Critical-period plasticity in the visual cortex. *Annu. Rev. Neurosci.* 35 309–330. 10.1146/annurev-neuro-061010-113813 22462544

[B63] Louis S. Gerstein G. L. Grun S. Diesmann M. (2010). Surrogate spike train generation through dithering in operational time. *Front. Comput. Neurosci.* 4:127. 10.3389/fncom.2010.00127 21060802 PMC2972681

[B64] Luhmann H. J. Khazipov R. (2018). Neuronal activity patterns in the developing barrel cortex. *Neuroscience* 368 256–267. 10.1016/j.neuroscience.2017.05.025 28528963

[B65] Luhmann H. J. Kilb W. Hanganu-Opatz I. L. (2009). Subplate cells: Amplifiers of neuronal activity in the developing cerebral cortex. *Front. Neuroanat.* 3:19. 10.3389/neuro.05.019.2009 19862346 PMC2766272

[B66] Luhmann H. J. Kirischuk S. Kilb W. (2018). The superior function of the subplate in early neocortical development. *Front. Neuroanat.* 12:97. 10.3389/fnana.2018.00097 30487739 PMC6246655

[B67] Luhmann H. J. Reiprich R. A. Hanganu I. Kilb W. (2000). Cellular physiology of the neonatal rat cerebral cortex: Intrinsic membrane properties, sodium and calcium currents. *J. Neurosci. Res.* 62 574–584. 10.1002/1097-4547(20001115)62:4<574::AID-JNR12>3.0.CO;2-0 11070501

[B68] Luhmann H. J. Schubert D. Kotter R. Staiger J. F. (1999). Cellular morphology and physiology of the perinatal rat cerebral cortex. *Dev. Neurosci.* 21 298–309. 10.1159/000017379 10575253

[B69] Luhmann H. J. Sinning A. Yang J. W. Reyes-Puerta V. Stüttgen M. C. Kirischuk S. (2016). Spontaneous neuronal activity in developing neocortical networks: From single cells to large-scale interactions. *Front. Neural Circuits* 10:40. 10.3389/fncir.2016.00040 27252626 PMC4877528

[B70] Mao T. Kusefoglu D. Hooks B. M. Huber D. Petreanu L. Svoboda K. (2011). Long-range neuronal circuits underlying the interaction between sensory and motor cortex. *Neuron* 72 111–123. 10.1016/j.neuron.2011.07.029 21982373 PMC5047281

[B71] Martini F. J. Guillamon-Vivancos T. Moreno-Juan V. Valdeolmillos M. Lopez-Bendito G. (2021). Spontaneous activity in developing thalamic and cortical sensory networks. *Neuron* 109 2519–2534. 10.1016/j.neuron.2021.06.026 34293296 PMC7611560

[B72] Matsubayashi Y. Iwai L. Kawasaki H. (2008). Fluorescent double-labeling with carbocyanine neuronal tracing and immunohistochemistry using a cholesterol-specific detergent digitonin. *J. Neurosci. Methods* 174 71–81. 10.1016/j.jneumeth.2008.07.003 18674563

[B73] McCormick D. A. Prince D. A. (1987). Post-natal development of electrophysiological properties of rat cerebral cortical pyramidal neurones. *J. Physiol.* 393 743–762. 10.1113/jphysiol.1987.sp016851 2895811 PMC1192421

[B74] McQuillen P. S. Sheldon R. A. Shatz C. J. Ferriero D. M. (2003). Selective vulnerability of subplate neurons after early neonatal hypoxia-ischemia. *J. Neurosci.* 23 3308–3315. 10.1523/JNEUROSCI.23-08-03308.2003 12716938 PMC6742293

[B75] Meng X. Kao J. P. Kanold P. O. (2014). Differential signaling to subplate neurons by spatially specific silent synapses in developing auditory cortex. *J. Neurosci.* 34 8855–8864. 10.1523/JNEUROSCI.0233-14.2014 24966385 PMC4069358

[B76] Meng X. Mukherjee D. Kao J. P. Y. Kanold P. O. (2021). Early peripheral activity alters nascent subplate circuits in the auditory cortex. *Sci. Adv.* 7:eabc9155. 10.1126/sciadv.abc9155 33579707 PMC7880598

[B77] Mezzera C. Lopez-Bendito G. (2016). Cross-modal plasticity in sensory deprived animal models: From the thalamocortical development point of view. *J. Chem. Neuroanat.* 75 32–40. 10.1016/j.jchemneu.2015.09.005 26459021

[B78] Miller R. (2000). *Time and the brain.* Amsterdam: Harwood Academic. 10.4324/9780203304570

[B79] Minamisawa G. Kwon S. E. Chevee M. Brown S. P. O’Connor D. H. (2018). A non-canonical feedback circuit for rapid interactions between somatosensory cortices. *Cell Rep.* 23 2718–2731.e6. 10.1016/j.celrep.2018.04.115 29847801 PMC6004823

[B80] Minlebaev M. Ben-Ari Y. Khazipov R. (2007). Network mechanisms of spindle-burst oscillations in the neonatal rat barrel cortex in vivo. *J. Neurophysiol.* 97 692–700. 10.1152/jn.00759.2006 17093125

[B81] Mizuno H. Ikezoe K. Nakazawa S. Sato T. Kitamura K. Iwasato T. (2018). Patchwork-type spontaneous activity in neonatal barrel cortex layer 4 transmitted via thalamocortical projections. *Cell Rep.* 22 123–135. 10.1016/j.celrep.2017.12.012 29298415

[B82] Molnár Z. Lopez-Bendito G. Small J. Partridge L. D. Blakemore C. Wilson M. C. (2002). Normal development of embryonic thalamocortical connectivity in the absence of evoked synaptic activity. *J. Neurosci.* 22 10313–10323. 10.1523/JNEUROSCI.22-23-10313.2002 12451131 PMC6758728

[B83] Molnar Z. Luhmann H. J. Kanold P. O. (2020). Transient cortical circuits match spontaneous and sensory-driven activity during development. *Science* 370 308–312. 10.1126/science.abb2153 33060328 PMC8050953

[B84] Moore A. R. Zhou W. L. Sirois C. L. Belinsky G. S. Zecevic N. Antic S. D. (2014). Connexin hemichannels contribute to spontaneous electrical activity in the human fetal cortex. *Proc. Natl. Acad. Sci. U.S.A.* 111 E3919–E3928. 10.1073/pnas.1405253111 25197082 PMC4169969

[B85] Moreno-Juan V. Filipchuk A. Anton-Bolanos N. Mezzera C. Gezelius H. Andres B. (2017). Prenatal thalamic waves regulate cortical area size prior to sensory processing. *Nat. Commun.* 8:14172. 10.1038/ncomms14172 28155854 PMC5296753

[B86] Myakhar O. Unichenko P. Kirischuk S. (2011). GABAergic projections from the subplate to Cajal-Retzius cells in the neocortex. *Neuroreport* 22 525–529. 10.1097/WNR.0b013e32834888a4 21666518

[B87] Nagode D. A. Meng X. Winkowski D. E. Smith E. Khan-Tareen H. Kareddy V. (2017). Abnormal development of the earliest cortical circuits in a mouse model of autism spectrum disorder. *Cell Rep.* 18 1100–1108. 10.1016/j.celrep.2017.01.006 28147267 PMC5488290

[B88] Nowak L. Bregestovski P. Ascher P. Herbet A. Prochiantz A. (1984). Magnesium gates glutamate-activated channels in mouse central neurones. *Nature* 307 462–465. 10.1038/307462a0 6320006

[B89] Paxinos G. (2007). *Atlas of the developing mouse brain : At E17.5, PO, and P6.* Amsterdam: Elservier.

[B90] Perreault P. Avoli M. (1989). Effects of low concentrations of 4-aminopyridine on CA1 pyramidal cells of the hippocampus. *J. Neurophysiol.* 61 953–970. 10.1152/jn.1989.61.5.953 2566657

[B91] Petersen C. C. (2007). The functional organization of the barrel cortex. *Neuron* 56 339–355. 10.1016/j.neuron.2007.09.017 17964250

[B92] Petersen C. C. H. (2019). Sensorimotor processing in the rodent barrel cortex. *Nat. Rev. Neurosci.* 20 533–546. 10.1038/s41583-019-0200-y 31367018 PMC7116865

[B93] Pires J. Nelissen R. Mansvelder H. D. Meredith R. M. (2021). Spontaneous synchronous network activity in the neonatal development of mPFC in mice. *Dev. Neurobiol.* 81 207–225. 10.1002/dneu.22811 33453138 PMC8048581

[B94] Preibisch S. Saalfeld S. Tomancak P. (2009). Globally optimal stitching of tiled 3D microscopic image acquisitions. *Bioinformatics* 25 1463–1465. 10.1093/bioinformatics/btp184 19346324 PMC2682522

[B95] Qu G. J. Ma J. Yu Y. C. Fu Y. (2016). Postnatal development of GABAergic interneurons in the neocortical subplate of mice. *Neuroscience* 322 78–93. 10.1016/j.neuroscience.2016.02.023 26892297

[B96] Richter D. Luhmann H. J. Kilb W. (2010). Intrinsic activation of GABA(A) receptors suppresses epileptiform activity in the cerebral cortex of immature mice. *Epilepsia* 51 1483–1492. 10.1111/j.1528-1167.2010.02591.x 20491873

[B97] Schindelin J. Arganda-Carreras I. Frise E. Kaynig V. Longair M. Pietzsch T. (2012). Fiji: An open-source platform for biological-image analysis. *Nat. Methods* 9 676–682. 10.1038/nmeth.2019 22743772 PMC3855844

[B98] Schlaggar B. L. O’Leary D. D. (1994). Early development of the somatotopic map and barrel patterning in rat somatosensory cortex. *J. Comp. Neurol.* 346 80–96. 10.1002/cne.903460106 7962713

[B99] Sheikh A. Meng X. Liu J. Mikhailova A. Kao J. P. Y. McQuillen P. S. (2019). Neonatal hypoxia-ischemia causes functional circuit changes in subplate neurons. *Cereb. Cortex* 29 765–776. 10.1093/cercor/bhx358 29365081 PMC6676960

[B100] Sieben K. Bieler M. Roder B. Hanganu-Opatz I. L. (2015). Neonatal restriction of tactile inputs leads to long-lasting impairments of cross-modal processing. *PLoS Biol.* 13:e1002304. 10.1371/journal.pbio.1002304 26600123 PMC4658190

[B101] Sohur U. S. Padmanabhan H. K. Kotchetkov I. S. Menezes J. R. Macklis J. D. (2014). Anatomic and molecular development of corticostriatal projection neurons in mice. *Cereb. Cortex* 24 293–303. 10.1093/cercor/bhs342 23118198 PMC3888374

[B102] Sreenivasan V. Esmaeili V. Kiritani T. Galan K. Crochet S. Petersen C. C. H. (2016). Movement initiation signals in mouse whisker motor cortex. *Neuron* 92 1368–1382. 10.1016/j.neuron.2016.12.001 28009277 PMC5196025

[B103] Staiger J. F. Petersen C. C. H. (2021). Neuronal circuits in barrel cortex for whisker sensory perception. *Physiol. Rev.* 101 353–415. 10.1152/physrev.00019.2019 32816652

[B104] Stüttgen M. C. Schwarz C. (2018). Barrel cortex: What is it good for? *Neuroscience* 368 3–16. 10.1016/j.neuroscience.2017.05.009 28526578

[B105] Suter B. A. Shepherd G. M. (2015). Reciprocal interareal connections to corticospinal neurons in mouse M1 and S2. *J. Neurosci.* 35 2959–2974. 10.1523/JNEUROSCI.4287-14.2015 25698734 PMC4331623

[B106] Sydnor V. J. Larsen B. Bassett D. S. Alexander-Bloch A. Fair D. A. Liston C. (2021). Neurodevelopment of the association cortices: Patterns, mechanisms, and implications for psychopathology. *Neuron* 109 2820–2846. 10.1016/j.neuron.2021.06.016 34270921 PMC8448958

[B107] Tiong S. Y. X. Oka Y. Sasaki T. Taniguchi M. Doi M. Akiyama H. (2019). Kcnab1 is expressed in subplate neurons with unilateral long-range inter-areal projections. *Front. Neuroanat.* 13:39. 10.3389/fnana.2019.00039 31130851 PMC6509479

[B108] Tiriac A. Rio-Bermudez C. Blumberg M. S. (2014). Self-generated movements with “unexpected” sensory consequences. *Curr. Biol.* 24 2136–2141. 10.1016/j.cub.2014.07.053 25131675 PMC4175005

[B109] Tolner E. A. Sheikh A. Yukin A. Y. Kaila K. Kanold P. O. (2012). Subplate neurons promote spindle bursts and thalamocortical patterning in the neonatal rat somatosensory cortex. *J. Neurosci.* 32 692–702. 10.1523/JNEUROSCI.1538-11.2012 22238105 PMC3517992

[B110] Valverde F. Facal-Valverde M. V. Santacana M. Heredia M. (1989). Development and differentiation of early generated cells of sublayer VIb in the somatosensory cortex of the rat: A correlated Golgi and autoradiographic study. *J. Comp. Neurol.* 290 118–140. 10.1002/cne.902900108 2480368

[B111] Viswanathan S. Bandyopadhyay S. Kao J. P. Kanold P. O. (2012). Changing microcircuits in the subplate of the developing cortex. *J. Neurosci.* 32 1589–1601. 10.1523/JNEUROSCI.4748-11.2012 22302801 PMC3517995

[B112] Viswanathan S. Sheikh A. Looger L. L. Kanold P. O. (2017). Molecularly defined subplate neurons project both to thalamocortical recipient layers and thalamus. *Cereb. Cortex* 27 4759–4768. 10.1093/cercor/bhw271 27655928 PMC6059176

[B113] Wallois F. Routier L. Heberlé C. Mahmoudzadeh M. Bourel-Ponchel E. Moghimi S. (2021). Back to basics: The neuronal substrates and mechanisms that underlie the electroencephalogram in premature neonates. *Neurophysiol. Clin.* 51 5–33. 10.1016/j.neucli.2020.10.006 33162287

[B114] Wess J. M. Isaiah A. Watkins P. V. Kanold P. O. (2017). Subplate neurons are the first cortical neurons to respond to sensory stimuli. *Proc. Natl. Acad. Sci. U.S.A.* 114 12602–12607. 10.1073/pnas.1710793114 29114043 PMC5703299

[B115] Woolsey T. A. Van der Loos H. (1970). The structural organization of layer IV in the somatosensory region (SI) of mouse cerebral cortex. The description of a cortical field composed of discrete cytoarchitectonic units. *Brain Res.* 17 205–242. 10.1016/0006-8993(70)90079-X 4904874

[B116] Workman A. D. Charvet C. J. Clancy B. Darlington R. B. Finlay B. L. (2013). Modeling transformations of neurodevelopmental sequences across mammalian species. *J. Neurosci.* 33 7368–7383. 10.1523/JNEUROSCI.5746-12.2013 23616543 PMC3928428

[B117] Xu X. Hanganu-Opatz I. L. Bieler M. (2020). Cross-talk of low-level sensory and high-level cognitive processing: Development, mechanisms, and relevance for cross-modal abilities of the brain. *Front. Neurorobot.* 14:7. 10.3389/fnbot.2020.00007 32116637 PMC7034303

[B118] Yamamoto N. Lopez-Bendito G. (2012). Shaping brain connections through spontaneous neural activity. *Eur. J. Neurosci.* 35 1595–1604. 10.1111/j.1460-9568.2012.08101.x 22607005

[B119] Yamashita T. Pala A. Pedrido L. Kremer Y. Welker E. Petersen C. C. (2013). Membrane potential dynamics of neocortical projection neurons driving target-specific signals. *Neuron* 80 1477–1490. 10.1016/j.neuron.2013.10.059 24360548

[B120] Yang J. W. An S. Sun J. J. Reyes-Puerta V. Kindler J. Berger T. (2013). Thalamic network oscillations synchronize ontogenetic columns in the newborn rat barrel cortex. *Cereb. Cortex* 23 1299–1316. 10.1093/cercor/bhs103 22593243

[B121] Yang J. W. Reyes-Puerta V. Kilb W. Luhmann H. J. (2016). Spindle bursts in neonatal rat cerebral cortex. *Neural Plast.* 2016:3467832. 10.1155/2016/3467832 27034844 PMC4806652

[B122] Zhao C. Kao J. P. Kanold P. O. (2009). Functional excitatory microcircuits in neonatal cortex connect thalamus and layer 4. *J. Neurosci.* 29 15479–15488. 10.1523/JNEUROSCI.4471-09.2009 20007472 PMC3539415

