# Supplementary figures and images for: Axonal connections between S1 barrel, M1, and S2 cortex in the newborn mouse
Source: Front Neuroanat. 2023 Jan 25;17:1105998. doi: 10.3389/fnana.2023.1105998 (PMC9905141; doi:10.3389/fnana.2023.1105998)

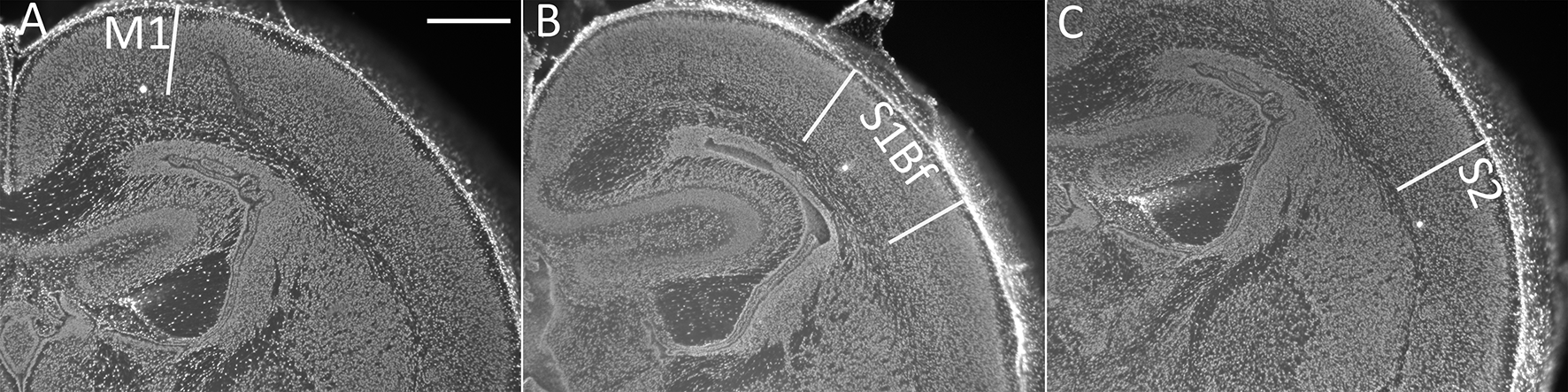

Supplement: Supplementary Figure 1 — Representative photomicrographs of DiI crystal injections at the SP-layer 6 border of M1 (A), S1Bf (B), and S2 (C) in coronal sections of P0 mouse brains. Immediately after DiI injection, photomicrographs were captured with an adequate fluorescence filter set for SYTOX™ Green nuclear marker and for DiI. White lines demark the DiI injection target areas from the adjacent sensory areas as estimated from reference points in the developing mouse brain atlas (Paxinos, 2007). Scale bar is 1 mm. [file Image_1.TIF]

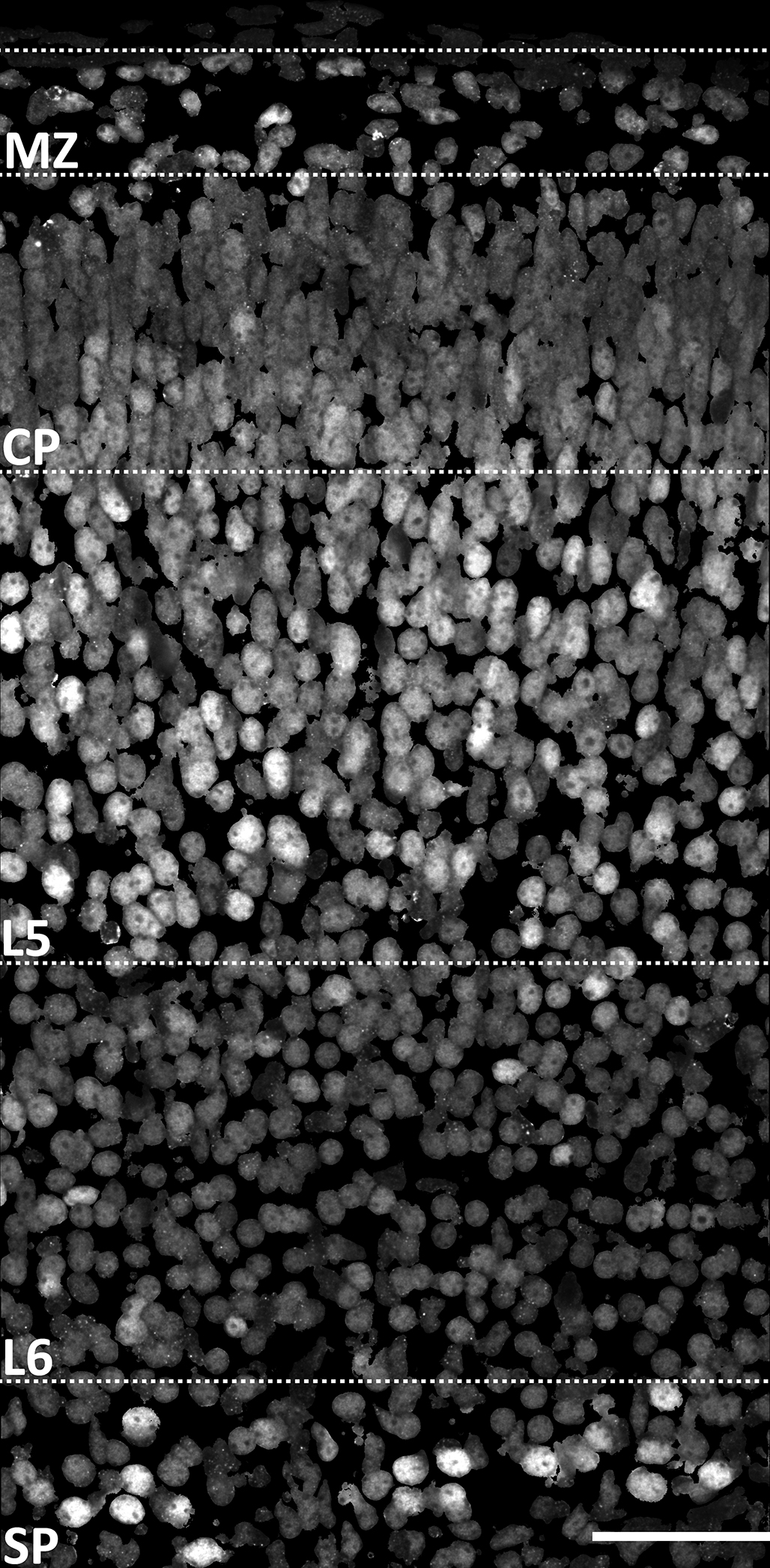

Supplement: Supplementary Figure 2 — Scheme for assigning the cortical layer boundaries and for determination of neuron number proportions across cortical layer to estimate expected backlabeled cell frequencies. Scale bar is 100 μm. [file Image_2.TIF]

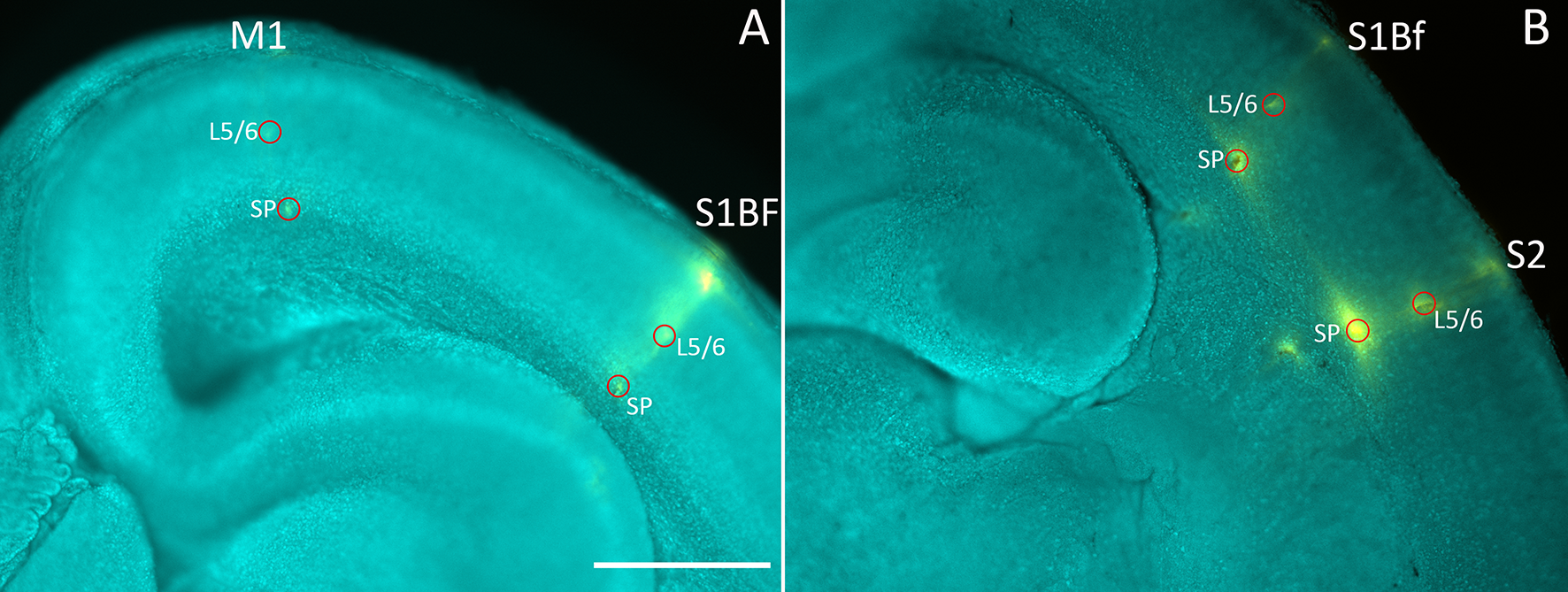

Supplement: Supplementary Figure 3 — Composite image of the DiI signal and the pseudocoloured DAPI signal showing silicon probe positioning in the M1 S1Bf panel (A) and S1Bf S2 panel (B) configuration. Position of the analyzed L5/6 and SP shanks is marked with red circles. Scale bar is 500 μm. [file Image_3.TIF]
